# Zero-deforestation commitments in Indonesia’s palm oil sector achieve high compliance but no additionality

**DOI:** 10.1073/pnas.2511503123

**Published:** 2026-07-13

**Authors:** Matthieu Stigler, Janina Grabs, Robert Heilmayr, Kimberly M. Carlson, Adelina Chandra, Jason Jon Benedict, Rachael D. Garrett

**Affiliations:** ^a^ Institute of Economics and Econometrics, Geneva School of Economics and Management, University of Geneva, Geneva 1211, Switzerland; ^b^ Department of Social Sciences, University of Basel, Basel 4056, Switzerland; ^c^ Environmental Studies Program and Bren School of Environmental Science and Management, University of California, Santa Barbara, CA 93106; ^d^ Department of Environmental Studies, New York University, New York, NY 10003; ^e^ Department of Environmental Systems Sciences, ETH Zurich 8092, Switzerland; ^f^ Department of Geography, Conservation Research Institute, University of Cambridge, Cambridge CB2 3QZ, United Kingdom

**Keywords:** sustainability standards, causal inference, zero-deforestation commitments, sustainable supply chains

## Abstract

In response to public pressure, companies have increasingly adopted voluntary sustainable sourcing commitments. Yet, it often remains unclear whether such commitments lead only to the narrow goal of supplier compliance, or also to the broader goal of additionality, benefits beyond what would have occurred without commitments. A company’s ability to influence its suppliers’ behavior partly depends on the complexity of supply chains, yet data on supply chain linkages are limited, inhibiting causal evaluations. Here, we evaluate the compliance and additionality of zero-deforestation commitments (ZDCs) in the Indonesian palm oil sector, a major driver of tropical deforestation. We compiled a detailed dataset that integrates information about corporate ZDC implementation, supply chain linkages, and maps of palm-driven deforestation in Indonesia. We use this dataset to evaluate the causal impact of ZDCs on deforestation in oil palm concessions from 2018 to 2020 using difference-in-differences econometric methods. We find that concessions linked to companies with ZDCs had low deforestation (<1% per year from 2018 to 2020) and thus largely achieved compliance. Yet they experienced similar reductions in deforestation compared to concessions not covered by ZDCs, therefore showing no additionality. We attribute our finding of no additionality to broader changes in policy and economic conditions during the study period that may have reduced the remaining accessible forest and dampened market and regulatory incentives to clear forests for palm oil. Should these conditions change in the future, continued compliance with ZDCs could yield additional reductions in deforestation.

## Significance

Despite public policy efforts, tropical deforestation continues at alarming rates. In response, companies have adopted zero-deforestation commitments (ZDCs) to eliminate deforestation from their supply chains. As ZDCs are transmitted to suppliers via complex supply chain linkages, understanding whether they really reduce deforestation requires a detailed understanding of such links. We leverage a unique dataset of sourcing relationships in the Indonesian palm oil sector to evaluate ZDC effectiveness. Our findings reveal that suppliers are complying with ZDCs. However, since both ZDC-linked and non-ZDC concessions experienced comparable deforestation reductions, we argue ZDCs have had no additional impacts. We attribute this to broader economic and policy conditions from 2018 to 2020 that reduced growers’ ability and incentives to clear forests for oil palm.

Tropical forests play a critical role for biodiversity, climate regulation, and carbon storage, but are disappearing at an alarming rate. Ninety percent or more of tropical deforestation between 2011 and 2015 was driven by agricultural commodities, notably beef, soy, and palm oil (1, 2). Recognizing that the processing and trade of deforestation-linked commodities is concentrated among a few large companies, supply chain-driven zero-deforestation commitments (ZDCs) have emerged as a promising complement to producer countries’ domestic policies to reduce deforestation. ZDCs are corporate pledges to refrain from producing or sourcing products grown on deforested land, typically after a cut-off date (3, 4). With the rise of due diligence regulations that ban deforestation-linked imports into the European Union and United Kingdom markets, zero-deforestation supply chains are rapidly becoming a legal, rather than voluntary, obligation for companies sourcing commodities such as palm oil, beef, or soy (5). Given the growing importance of supply chain regulations governing deforestation, evaluating the effectiveness of voluntary ZDCs at stemming forest loss is an important precondition for understanding whether existing efforts are meeting the scale of our global forest conservation challenge.

Evaluating the effectiveness of ZDCs requires assessing not just compliance—whether producers in ZDC supply chains adhere to companies’ policies and avoid deforestation—but also additionality—whether ZDCs lead to lower rates of deforestation than would have occurred in their absence. Compliance can generally be interpreted as success from a corporate perspective, since the company achieved its goal of ensuring a deforestation-free supply chain. However, during periods of systematically low deforestation, most producers are likely to be deforestation-free. Low deforestation helps supply chain actors comply with ZDCs, but it can also lead actors with ZDCs to overstate claims of additionality. Therefore, the mere presence of compliance does not establish the additionality of deforestation reductions attributable to ZDCs, calling for a separate analysis of additionality.

However, assessing the additionality of supply chain policies presents significant challenges. Impact evaluations aim to tease out the causal effect of a policy by identifying who has been treated by the policy and comparing the effects on this group to a counterfactual of what would have happened in the absence of the policy (6, 7). While such analyses are well understood when there is a clearly defined treatment group, they become more methodologically challenging for the study of supply chain policies for several reasons. First, supply chains are composed of multiple layers of actors, starting with agricultural producers, passing through processors such as mills, refineries, and slaughterhouses, and finishing with traders, exporters, retailers, and consumers. Even when it is known which downstream corporate actors adopted and implemented a ZDC and are therefore treated, it is often unclear which upstream producers are treated due to the multiple and often interwoven linkages between downstream companies and producers (e.g., in a given year, one producer may sell to several companies and each of these companies may buy from hundreds of producers). Second, different types of linkages exist: Supply chain actors can be linked to each other either through legal ownership or commercial sourcing relationships. This implies that companies have different channels to implement their ZDCs and that multiple types of treatment must be considered. Third, these ownership or sourcing relationships may change over time—depending on the degree of supply chain stickiness (8)—further complicating the identification of treated units. Thus, determining treatment status in supply chains requires temporally resolved data on ownership and sourcing linkages between all actors. Yet, despite multiple calls for transparency and traceability, the supply chain data needed to assess ZDCs has only recently become available (9).

Indeed, the prior absence of such data has limited evaluation of ZDCs in the palm oil sector, which has driven rapid deforestation in Southeast Asia (10–13). The sector has comparatively high adoption of corporate ZDCs, whose implementation became effective in 2018 (14). By 2020, ZDCs covered roughly 83% of palm oil refining capacity in Indonesia and Malaysia (15). In Indonesia, the world’s leading palm oil producer and country with one of the highest rates of primary forest loss in the tropics, oil palm expansion accounted for around 34% of all deforestation from 2001 to 2019 (13). Yet, studies evaluating palm oil ZDCs have remained limited to ex-ante assessments on biodiversity (16, 17) and deforestation (18–20) from coarser-scale data.

Ex-post impact evaluation studies in the palm oil sector have primarily looked at the effects of certification under the Roundtable on Sustainable Palm Oil (RSPO). The RSPO is the main sustainability certification system in the sector and has prohibited clearance of High Conservation Value lands since 2005. Strong zero-deforestation criteria (i.e., identification and conservation of High Carbon Stock areas) have been part of the standard only since late 2018 (21–29). While RSPO certification has reduced deforestation (21, 24, 25), this impact occurred mainly on plantations with little remaining forest (a targeting challenge) (21), and deforestation reductions were offset by deforestation leakage to land outside of Indonesia’s State Forest (a spillover challenge) (24). Furthermore, fires, forest loss, and degradation continued even within certified plantations (22, 23, 27). In the case of the RSPO, the treatment status identification challenge is relatively easily addressed as certification and RSPO membership provide a clear definition of treated (i.e., plantations owned by RSPO members or certified by the RSPO) and nontreated (i.e., noncertified or nonmember plantations) units. As such, available RSPO studies inform us about whether oil palm growing companies were able to reduce deforestation within plantations they owned and/or certified (21–28) through third-party audits (30). They do not provide insight into whether supply chain companies were able to reduce deforestation among all the plantations they source from, regardless of ownership, through implementation of their ZDCs. Indeed, understanding whether a company can exert sufficient leverage as a buyer to influence its suppliers’ practices, or if it needs instead to acquire direct ownership to ensure their compliance, is a crucial question regarding the (vertical) organization of sustainable supply chains, yet has been left unanswered. Given the targeting and spillover challenges of mill-level certification, together with the limited coverage of RSPO-certified palm oil [about 19 to 20% of global production, (31)], there is a need for broader analyses documenting whether companies are fulfilling their ZDC pledges, and if such compliance reduces deforestation in both their own and suppliers’ plantations.

To date, evaluation of ZDCs has primarily focused on cattle (32–36) and soy (37–39) in Brazil and timber in Chile (40). In Brazil, the Soy Moratorium and cattle ZDCs have contributed to deforestation reductions (36, 38), though their impact would have likely been even larger if more companies had participated (36, 38), if the policies were fully implemented (34, 37), and if laundering and leakage opportunities were reduced (32, 33, 35, 39, 41). To link treated companies to their treated suppliers, these studies usually face a trade-off between the precision and number of linkages considered: They must either focus on well-defined company-supplier linkages and thus cover only a small number of actors or regions (33–35), or consider a large number of suppliers but use only a proxy of their linkages to companies such as the distance to a slaughterhouse (32) or the share of committed slaughterhouses in a given municipality (36, 37).^*^

Here, we combine recently available data detailing the Indonesian palm oil supply chain with assessments of company-level ZDCs and remotely sensed measures of oil palm-driven deforestation inside Indonesian oil palm concessions during the 2001–2020 period. This unique dataset allows us to conduct causal evaluations of the effects of palm oil ZDCs in Indonesia on forest loss and make several contributions to the literature. First, compared to previous research focusing on RSPO in Indonesia, we investigate whether ZDC implementation by oil palm companies reduced deforestation among all of the owned or supplying plantations that can be linked to them rather than among only a (possibly selective) subset of their RSPO-certified plantations. Second, compared to prior ZDC studies, we overcome the trade-off between the precision and number of linkages since we can analyze detailed supply-chain linkages while covering nearly all Indonesian plantations that could be linked downstream. Finally, the availability of data on both ownership and sourcing links between companies and their suppliers allows us to compare the relative effectiveness of ownership versus sourcing pathways in influencing deforestation.

We aim to assess the effectiveness of corporate ZDCs in the oil palm sector in reducing deforestation in Indonesia implemented through ownership and sourcing pathways. Specifically, we ask four questions: 1) How complex is the Indonesian palm oil supply chain and how does this complexity impact the identification of ZDC-linked concessions? 2) How compliant (i.e., degree to which zero-deforestation was achieved) were companies with ZDCs? 3) How much additionality (i.e., reductions in deforestation beyond what would be expected without ZDCs) was achieved by companies’ ZDCs? 4) Were ZDCs with sourcing-based linkages as compliant and additional as those with ownership-based linkages?

## 1. Results

### 1.1. Supply Chain Linkages Are Complex, Overlapping and Show Moderate Persistence over Time.

**Fig. 1.**
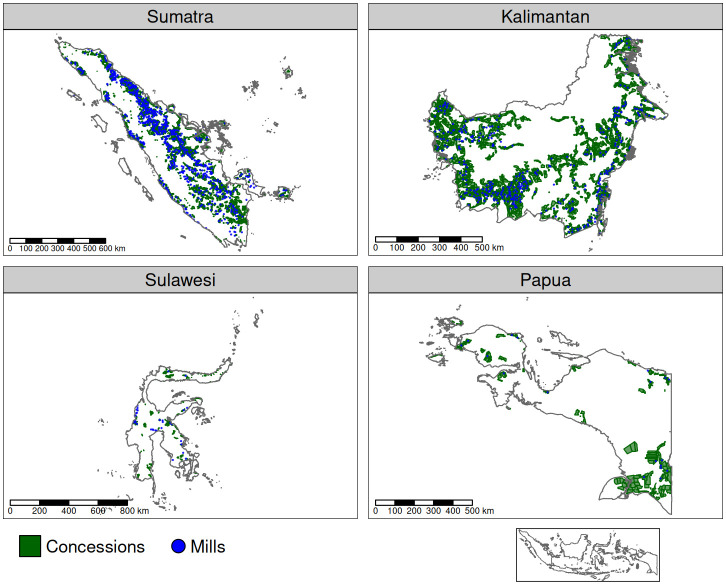
Oil palm concessions (green) and palm oil mills (blue) located in the main palm-oil producing islands.

Our dataset, which provides information about supply-chain linkages in the palm oil sector between 2018 and 2020 (*Data Description*), reveals the commonly described funnel-shaped structure of supply chains from producer to refiner (42, 43). At each stage of the chain, we observe a decreasing number of actors (Fig. 1): 2,644 oil palm concessions, 1,222 palm oil mills owned by 190 company groups (hereafter mill-owning companies) and 75 palm oil refineries owned by 32 refining company groups (hereafter refining companies).

**Fig. 2.**
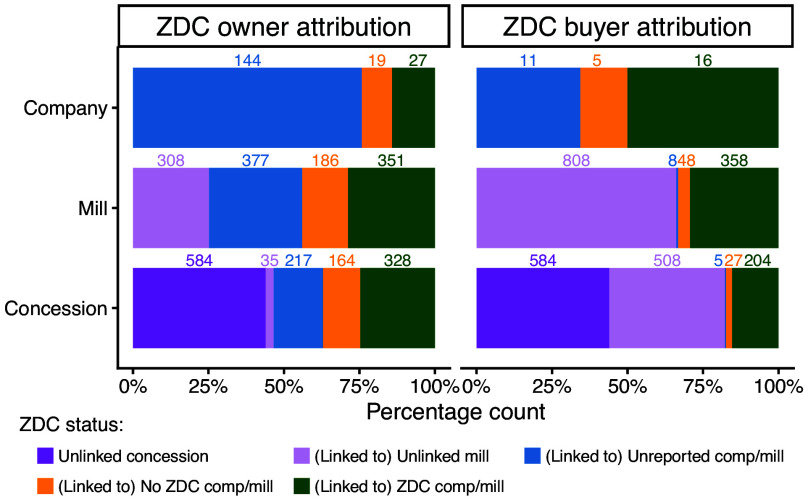
ZDC status across supply chain levels by attribution method. Company groups (*Top* row) may influence their supply chain through mill ownership linkages (*Left* column) or sourcing linkages (*Right* column), potentially changing practices of both mills (*Middle* row) and their supplying concessions (*Bottom* row).

We attempted to link concessions via mills to company groups using two approaches: 1) their linkages to mill-owning companies (*ownership-based attribution*) and 2) their most dominant sourcing linkages with refining companies (*buyer-based attribution*) derived from traceability reports (*Data Description*). We identified reports for 24% and 66% of owner and refining company groups; linked 75% of mills to a mill-owning company group and 34% to refining company groups; and linked 56% of concessions to mills (Fig. 2). Missing reports or linkages propagated into lower levels, with 31% and 1% of mills linked to a company without a report, 16% and 0.4% of concessions linked to a mill itself linked to unreported companies (*linked to unreported*), and 3% and 38% of concessions linked to an unlinked mill (*linked to unlinked*) according to the owner- and buyer-based linkages, respectively.

Mill-owning companies hold an average of 4.3 mills annually, while refining companies transact with an average of 138 distinct mills per year. These linkages introduce cross-relations between mill-owning and refining companies. On average, a refining company buys annually from 35.5 distinct mill-owning companies, whereas a mill-owning company sells annually to 3.1 refining companies. Conversely, an average individual mill sells to 2.2 sourcing companies each year. Calculating these statistics at the concession level is more complicated due to limited information linking concessions and mills. However, an analysis of RSPO-certified mills with public sourcing information indicates that an average mill sources from a median of three distinct industrial plantations per year.

We find that the linkages between refining companies and individual mills changed frequently over time between 2018 and 2020: The stickiness coefficient, a measure of the persistence of linkages over time (8), is 0.47. This indicates relatively low stickiness, noting that there is only a 47% probability that a linkage between a refining company and a mill persists from one year to the next. Furthermore, only 54% of observed linkages persisted over the three-year period covered by our data, whereas 26% of linkages appeared only for one year. On the other hand, ownership linkages are much more persistent, with a stickiness coefficient of 0.96 and 91% of linkages between mills and their owning companies persisting over three years.

### 1.2. ZDC Coverage Through Refinery Sourcing Relationships Far Exceeds Coverage Through Mill Ownership Relationships.

To identify the coverage of ZDCs across the supply chain and subsequently identify ZDC-linked concessions, we first classified mill-owning and refining companies as “No ZDC” or “ZDC” based on the strength of their public commitment reports, or “unreported” if no ZDC information was available. We then attributed a ZDC status to every linked mill and subsequently to every linked concession (Fig. 2) using the ownership- or sourcing-based attribution described above.

Public reports were identified for 24% of mill-owning companies (*Top Left*) and for 66% of refining companies (*Top Right*). Among all mill-owning company groups, 14% have a ZDC, while 50% of the refining company groups have a ZDC. Using ownership or sourcing linkages to attribute a score to mills and then to concessions, we find substantial differences in ZDC coverage between ownership and sourcing approaches. Based on ownership, 38% of linked mills and 46% of linked concessions have ZDCs. When considering sourcing, 86% of linked mills as well as 86% of linked concessions are covered by ZDCs. The ownership- and sourcing-based attribution approaches identify a different set of concessions covered by ZDCs: Only 48% of concessions have the same ZDC status according to both the ownership and sourcing attribution methods (*SI Appendix*,Table S18).

### 1.3. High Deforestation Rates Before ZDC Declaration Were Followed by Declining and Then Low Deforestation During Implementation.

**Fig. 3.**
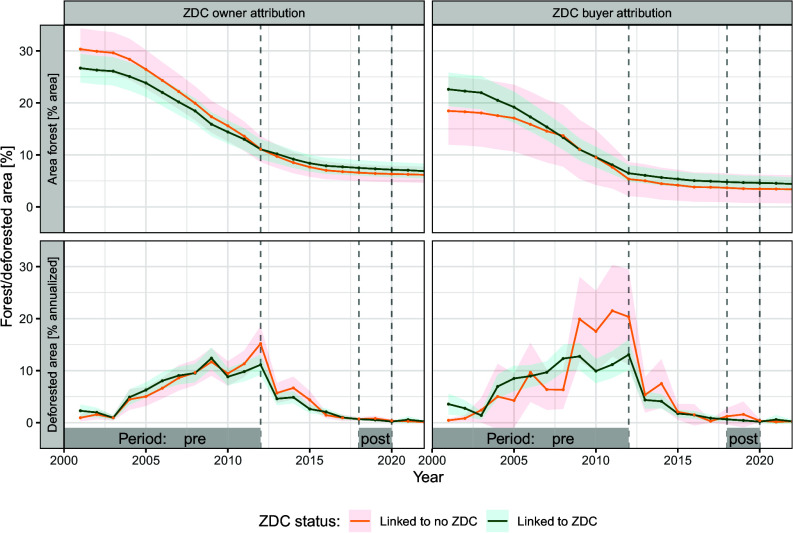
Forest cover and deforestation rates within concessions averaged by ZDC status. Vertical dashed lines represent the periods used for the causal analysis: the “pre” period runs from 2001 to 2012 and the “post” from 2018 to 2020. Shaded areas represent 95% CIs of the averages.

Turning to the average deforestation rates among the two main groups of interest, the *no-ZDC* and *ZDC* concessions, we find high annual rates of oil palm-driven deforestation peaking above 10% during the 2005–2012 period, followed by a rapid decline during 2013–2017 and then stabilization at low rates below 1% during 2018–2022 (Fig. 3).

These time periods overlap with the introduction and implementation of ZDCs (*SI Appendix*, Fig. S1). In 2011–2012, at around the time deforestation rates peaked in concessions, the first companies began publishing initial no-deforestation pledges. Most company groups adopted their commitments between 2013–2014. Implementation–the execution and enforcement of ZDCs—started in around 2014, and by 2018, when annualized deforestation was 1.4%, all the companies with ZDCs in our database were implementing their pledges (14, 44). We therefore refer to 2001–2012 as the “before ZDC implementation” period, 2013-2017 as “partial ZDC implementation,” and 2018 onward as the “full ZDC implementation” period.

Contrasting forest cover and deforestation trends across ZDC categories, we observe two key patterns: 1) a reduction in the spread between ZDC and no-ZDC concessions over time, where initial differences in both forest cover and deforestation rates tend to converge toward very similar levels after 2018, and 2) a relatively small difference in 2001 forest cover in ZDC compared to non-ZDC concessions, with the relative ranking between them changing based on whether the definition is sourcing- or owner-based. This initial difference is much smaller compared to the *unlinked* and *linked to undetermined* groups (*SI Appendix*, Table S1 and Fig. S4) as well as compared to the 16% gap between RSPO-certified and noncertified groups reported in ref. 21. This small difference indicates that the non-ZDC control group is similar to the treated ZDC group in terms of average land cover and land cover change, long before companies made their pledges. It also suggests that ZDC companies did not necessarily choose which concessions to own or source from based on initial forest cover, a key feature for our subsequent analysis using non-ZDC concessions as a control group.

### 1.4. Companies Collectively Achieved High Compliance with ZDCs from 2018 to 2020.

To investigate compliance, i.e. the degree to which ZDCs were associated with zero deforestation, we examine deforestation rates in ZDC-linked concessions for the period after the policies were implemented when we have reliable supply chain data (2018–2020). We focus on industrial palm-driven deforestation, as it is primarily influenced by palm oil company groups and represents the dominant and most reliably measured source of deforestation in concessions.^†^ We find that average annualized deforestation rates in concessions linked to ZDCs via both owner- and sourcing-based attributions were low in 2018–2020, both around 0.5% y^-1^, in sharp contrast with rates of 7 to 8% y^-1^ before 2012 (Table 1). These low deforestation rates during the “full implementation” period indicate high compliance with ZDCs on average. Nevertheless, from 2018–2020, about 3% of ZDC concessions in both owner- and sourcing-based attributions had mean deforestation rates with respect to 2000 forest cover above 1% y^-1^, while total deforestation across all ZDC concessions amounted to about 7,000 ha. Considering compliance over the “partial ZDC implementation” period (2013–2020), we still find relatively low rates of average deforestation around 1.2% y^-1^. Over this longer period around 8.8% of ZDC concessions had mean deforestation rates exceeding 1% y^-1^, suggesting companies collectively and gradually reached high compliance by 2020.

**Table 1.** Results of the difference-in-differences analysis

	ZDC owner attribution		ZDC buyer attribution	
ZDC group:	No ZDC	ZDC	No ZDC	ZDC
*N*	164	328	27	204
Mean pre:	7.12***	7.10***	9.56***	8.42***
Mean post:	0.62	0.48	1.06	0.42
Diff post-pre:	−6.50***	−6.63***	−8.50***	−8.00***
Diff-diff:	−0.12 (0.67)		0.49 (1.44)	
Wald stat:	0.49		<0.01	

***P < 0.001; **P < 0.01; * *P* < 0.05; and · *P*< 0.1. SEs clustered at the concession level. Outcome variable: Deforested area [% annualized]. *pre* denotes the 2001–2012 “before ZDC implementation” period and *post* the 2018–2020 “full ZDC implementation” period. For the corresponding DiD regression table, including the total number of observations, see *SI Appendix*, Table S2.

### 1.5. ZDCs Did Not Yield Additional Deforestation Reductions.

To investigate additionality, i.e. the additional effect of ZDCs on deforestation rates beyond general trends, we use a difference-in-differences (DiD) causal inference approach comparing the deforestation means of treated (ZDC) versus control (*no* ZDC) concessions before ZDC pledges (2001–2012) and during (2018–2020) full ZDC policy implementation.

When defining these groups based on mill ownership linkages, we find that they experienced very similar reductions in deforestation of −6.50 (ZDC) and −6.63 (no ZDC) percentage points (see row *Diff post-pre* in Table 1). The difference-in-differences coefficient, equal to the difference (ZDC versus non-ZDC) between the two temporal differences (*pre* versus *post*), confirms these comparable trends in deforestation. ZDCs were associated with a small and nonsignificant decrease in annualized deforestation rates of −0.12 percentage points. The Wald test of parallel pretrends is not rejected (see last row of Table 1), increasing confidence that the DiD model provides a causal estimate of the effect of ZDCs for the owner-based ZDC attribution.

Turning to the sourcing-based attribution, changes in deforestation rates are also similar across non-ZDC and ZDC concessions. The DiD coefficient is positive (0.48) yet not significant. However, the two groups did not exhibit parallel pretrends, and the low stickiness of sourcing-based linkages raises the possibility of underestimation bias, as some partially treated units may have been considered as controls (*Materials and Methods*). Together, these concerns warrant caution in drawing causal interpretations from this DiD model.

We interpret these DiD results, in particular those using the owner-based attribution, as evidence of a lack of additionality of ZDCs: Similar reductions in deforestation rates were observed in both the ZDC and non-ZDC groups. Given nonsignificant results for both the owner- and sourcing-based approaches, we cannot compare whether ownership-based linkages are more effective at reducing deforestation than sourcing-based linkages.^‡^

**Fig. 4.**
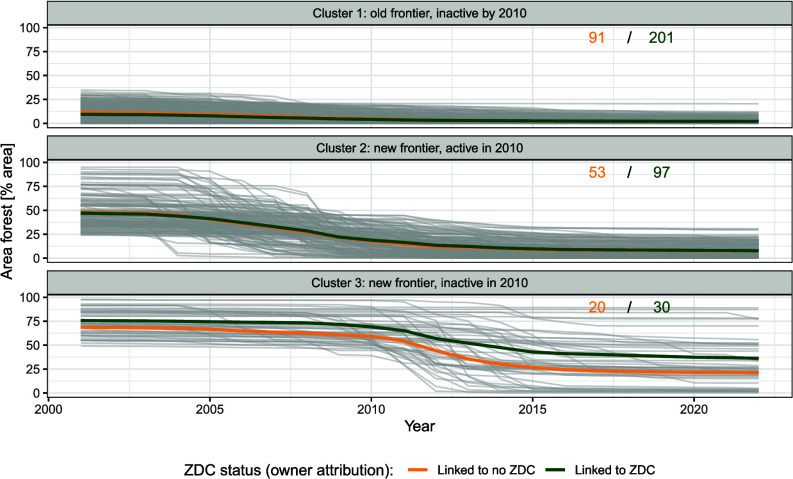
Forest cover by cluster for each concession (gray lines), together with the average forest cover across ZDC and non-ZDC concessions based on the owner attribution. For an interpretation of the clusters established using year 2000 and 2010 forest cover, see details in *SI Appendix*, section C.2.

A concern with the use of a DiD model is that companies might select the concessions they own or source from based on the concessions’ previous deforestation patterns, which could potentially invalidate the parallel trend assumption. For example, ref. 21 show that concessions certified by the RSPO had more oil palm and less forest in 2000, compared to other concessions. Furthermore, as Garrett et al. (45) argue, potential additionality from ZDC policies is higher when there are higher deforestation threats, which vary across frontier stages (46), suggesting heterogeneity in the DiD coefficients across frontier stages. To address the first point, we investigate whether concessions’ ZDC status can be explained by variables such as forest cover in 2000 and 2012, concession area, distance to mill, or average deforestation rate in the 2001–2012 period. The analysis reveals that only a few variables are significantly related to ZDC status, and that these variables have very low explanatory and predictive power (*SI Appendix*, Table S15). Furthermore, conducting our DiD analysis after matching on these variables still yields the finding of no additionality (*SI Appendix*, Fig. S6). To address the second point, we cluster our concessions based on their forest cover in 2000 and 2010 (see Fig. 4 and details in *SI Appendix*, section C.2) and conduct a DiD within each cluster. The clusters indicate that most of the concessions included in our analysis (both ZDC and non-ZDC) are “old frontiers” with low to zero deforestation. Few concessions had high remaining forest area in 2010. The DiD coefficients are not significant in most clusters (*SI Appendix*, Table S3), suggesting selection on forest cover and deforestation rates is not a reason for the null additionality results. We find only one exception with a significant coefficient yet a strong rejection of the parallel pretrend test, suggesting caution in interpreting this result as causal.

The previous analysis was conducted using the *no-ZDC* concessions as a control group for the *ZDC* concessions. This choice was motivated by the higher uncertainty in attributing a ZDC status to the *unlinked* or *linked to undetermined* concessions, and because the *no-ZDC* group followed a trajectory more similar to the *ZDC* group during the pre-ZDC period (*SI Appendix*, Fig. S4 and Table S1). However, because the higher uncertainty comes from either 1) the absence of information linking concessions to mills (*unlinked*), or 2) linkages to mills whose ZDC status itself could not be determined (*linked to undetermined*), there is still a high likelihood that *unlinked* and *linked to undetermined* concessions were not subject to ZDCs. We therefore reran our DiD analysis using these alternative groups as controls. Results, shown in *SI Appendix*, Table S4, reveal a trade-off between strength of the results and credibility of the analysis: We find either weak (small and nonsignificant DiD coefficients) and more credible (lack of rejection of the parallel pretrend test) results, or strong (large and significant coefficients) yet less credible (rejection of the parallel pretrend test) results. In general, we believe these results reinforce our broader conclusion that ZDCs have not caused additional declines in deforestation.

Finally, we conduct a variety of robustness tests and re-estimate our DiD models 1) within individual islands (*SI Appendix*, Table S5), 2) changing the pre- or post-periods in various ways, in particular considering the full 2013–2020 period as the treated period (*SI Appendix*, Table S6), 3) further disaggregating the *ZDC* category into *low* and *high* ZDC commitments (*SI Appendix*, Table S7), 4) using alternative definitions of deforestation (*SI Appendix*, Table S8) or using forest cover (*SI Appendix*, Table S9), 5) comparing industrial-palm-driven deforestation to any deforestation in *SI Appendix*, Table S10, 6) using an event-study analysis in *SI Appendix*, Fig. S5, 7) using a propensity-score matched DiD in *SI Appendix*, Fig. S6, 8) using alternative estimators such as matrix completion (47) or generalized synthetic control (48) in *SI Appendix*, Table S11, 9) looking at annual variations in treatment using (49) in *SI Appendix*, Fig. S7, 10) using only concessions persistently linked to ZDCs in *SI Appendix*, Table S13, 11) using as control concessions only those classified as control under both the owner- and sourcing-based ZDC scores in *SI Appendix*, Table S14, and 12) changing the sample inclusion rules (*SI Appendix*, Table S12). These additional tests largely confirm our main finding of no effect of ZDCs on forest area and deforestation, except for a few cases that are not unexpected given the large number of hypothesis tests conducted.

## 2. Discussion and Conclusion

In media, corporate press releases, and civil society organization reports, zero-deforestation commitments by companies in the palm oil sector have been variously framed as grand successes or abject failures (50–52). The truth behind these headlines depends on how one defines the goals and measures the impacts of such policies. Seen only through a before-and-after lens, Indonesian palm oil ZDCs appear to be highly successful at reducing deforestation (53). However, our analysis, using a counterfactual approach, indicates that ZDC supply chains did no better than non-ZDC supply chains in terms of deforestation. On average, all concessions experienced substantially reduced levels of deforestation after 2012, especially compared to the forest loss peak in around 2010. During the *full ZDC implementation* period (2018–2020), deforestation rates in both the treated and control groups were about 0.5% y^-1^, much lower than the approximately 7% y^-1^ observed during the *before ZDC implementation* period 2001–2012. Thus, ZDC firms appear to be broadly complying with their own policies (a success from an internal corporate perspective), but these policies are not generating additional avoided deforestation. In the relatively low-deforestation environment of Indonesia in the assessed period, it is thus difficult to gauge the broader policy effectiveness of ZDCs, and whether they were able to significantly curtail high deforestation pressure.

Why do we observe high compliance without additionality? We posit that our result stems from a combination of low access to remaining forests, and decreasing market and regulatory incentives to clear forests. First, our analysis shows that companies had **low access to remaining forests to clear** within allocated concessions, where most forests had already been cleared by 2012. On average, ZDC concessions lost 50% of their year 2000 forest during the 2001–2012 period, and by 2012 the remaining forest covered, on average, only 11% of the concessions’ area. This result mirrors the findings from ref. 21 which found that RSPO certification in the Indonesian palm oil sector was awarded to concessions largely free from forest. Interestingly, non-ZDC concessions displayed a similar pattern, with an average loss of 54% of 2000 forest and a remaining forest cover of 10% in 2012. Taken together, these two observations suggest possible explanations for why we see compliance (i.e., lack of remaining forest in ZDC concessions) and a lack of additionality (i.e., a similarly low forest area in non-ZDC concessions). However, ZDC implementation may have had impacts on forest outside of concessions in our dataset. There is an increasing concern about off-concession deforestation, undertaken by industrial companies, mid-sized actors working without concession permits, and smallholder farmers (14). This is partly corroborated by ref. 24 who find that the proximity to RSPO-certified mills had both positive and negative effects on deforestation outside concessions depending on the type of land zoning considered.

A second contributing factor may be that, during the 2018–2020 treatment period, companies had decreasing and relatively **low market incentives to clear** remaining forested land within concessions. Crude palm oil prices were significantly lower from 2018 to 2020 compared to 2001 to 2012 (*SI Appendix*, Fig. S3), reducing the likelihood of achieving positive returns on palm investments during 2017–2020 (14). Gaveau et al., in analyzing the rise and fall of forest loss in Borneo (54) and Indonesia as a whole (13), find that the price of crude palm oil is positively correlated with plantation expansion in the following year. Similarly, both refs. 55 and 56 find a sizable causal effect of prices on deforestation, further supporting this argument. While palm oil prices started to increase in 2021, deforestation did not increase in the subsequent year (13), though there may be lags between price changes and oil palm development given the long pause in clearing and associated higher start-up costs.

Finally, the introduction and implementation of ZDCs coincided with multiple state-led moratoria which potentially decreased companies’ **regulatory incentives to clear**. In 2011, Indonesia instituted a moratorium on the allocation of new forestry, agriculture, and mining concessions, including oil palm concessions, in primary forest and peatlands (57). This was followed by a nationwide ban on clearing carbon-rich deep peatlands in 2016 (58) and a 2018 to 2021 moratorium on new oil palm plantation permits (59). These public policies decreased companies’ ability to establish and exploit new concessions, especially in areas with high forest cover, potentially explaining the low deforestation rates we observe across both ZDC and non-ZDC concessions. There are, however, two caveats to this argument. First, these policies have been criticized as ineffective by civil society and media observers due to the existence of loopholes, as well as a lack of transparency, monitoring, and enforcement (60, 61). Scholarly work questions the effectiveness of the forest protection moratorium in significantly reducing forest and peatland loss compared to control areas (57, 62). Second, the ban on new concessions pertains to concessions that are, by definition, not included in our sample of already-allocated concessions. While we expect that in the absence of this ban, hypothetical newly allocated concessions would have had more forest and therefore experienced higher deforestation when developed for oil palm, providing a greater opportunity for ZDCs to conserve forest and achieve additionality, this counterfactual scenario is challenging to evaluate in practice.

In sum, our finding that ZDCs lacked additionality must be interpreted within the specific context of relatively low 2018–2020 deforestation rates across Indonesia. However, should access to forests, market incentives, and regulatory policies change and increase deforestation among some palm oil producers, ZDCs could achieve additionality provided that producers covered by ZDCs maintain compliance with these corporate policies. As such, we hypothesize that the additionality of ZDCs might become more significant during deforestation peaks than during the relatively calm period observed here (29, 45). This echoes the literature on the interactions between public and private conservation policies (63–65), in particular the hypothesis made by ref. 66 that “transnational actors provide a bulwark against changes in political winds.” Only future developments, possibly triggered by increases in international prices or policies that create local demand such as biofuel mandates (67), will allow researchers to test this hypothesis.

Unlike most prior work, our study defines ZDC treatment based on observed supply-chain linkages rather than proxies such as distance to processing facilities. In doing so, we were able to study whether companies, through ownership or sourcing channels, can transmit their sustainability pledges to their suppliers. Yet, our findings highlight how supply chain dynamics can complicate impact evaluations of supply-chain policies such as ZDCs. The complexity we documented, both in terms of interdependence and low stickiness of linkages, suggests that distinguishing between treated and nontreated units will remain a challenging task. This is exemplified by our owner- and sourcing-based attributions of ZDC status identifying different sets of concessions, with only 48% overlap under both definitions. Furthermore, the fact that we could not observe supply-chain linkages during the *partial implementation* period raises the possibility that some concessions classified as controls in 2018–2020 were in fact part of the *treated* supply chain during 2013–2017, potentially leading to an underestimation bias. The low stickiness of sourcing-based linkages makes this scenario more plausible, suggesting that results relying on these linkages should be interpreted with caution.

The complexity of the supply chain also introduces the possibility that positive spillovers—unintended and indirect benefits that transfer from treated to nontreated units—occurred. Given the low stickiness and interdependence of trade linkages, an oil palm grower might perceive the risk of clearing land and thereby cutting itself off from potential buyers as too high to be worth it, even if the grower is currently not under any ZDC obligation. Furthermore, NGO watchdog activities and initiatives advocating for collective action across the entire sector (e.g. the Consumer Goods Forum) might also affect oil palm growing companies that are not directly linked to ZDCs (4). If this is true, such positive spillovers may contribute to our finding of no additionality, as nontreated units might have mimicked treated units. If this is the case, our analysis underestimates the effectiveness of ZDCs. While we cannot totally rule out this possibility, the analysis using alternative concession groups that are less likely to be affected by spillovers as controls either confirmed the finding of no additionality or proved inconclusive (*SI Appendix*, Table S4).

By shedding light on the additionality of supply chain policies in complex and dynamic supply chain structures and contexts, our results directly inform new deforestation and due diligence supply chain policies being set or discussed in Europe. In particular, our work informs ex-ante understanding of the potential impacts of the EU Deforestation Regulation, which aims to reduce the EU’s carbon emissions footprint by prohibiting the import of deforestation-linked commodities including palm oil. Our results demonstrate that the vast majority of industrial palm oil production in Indonesian concessions is likely to meet the EUDR zero-deforestation requirement as the associated land was cleared much earlier than the EU’s 2020 cut-off date. On the other hand, our work also shows that this state of affairs is based on large-scale clearance of forests prior to 2020, and that more recent lower deforestation may be due to favorable market and regulatory incentives that allowed for a substantial decline in industrial oil palm-driven deforestation. The low degree of purchasing stickiness and high number of overlapping linkages between sourcing companies documented here suggests that, at present, establishing completely differentiated ZDC supply channels may require substantial changes to sourcing strategies (e.g., reducing the number of suppliers) so that companies selling to the EU can avoid litigation risks related to the EU Deforestation Regulation. This outcome in turn runs the risk that companies consolidate their supply chains vertically, excluding smallholder producers (43, 68–72).

More broadly, our research adds to a growing body of evidence suggesting that tackling deforestation through supply chain policies often has limited direct effects on deforestation. A similar result was found for beef, the largest deforestation risk commodity in Brazil (36). In cases where voluntary supply chain policies have generated greater additionality, such as the Soy Moratorium in Brazil (37) and RSPO-certified oil palm plantations in Indonesia (21), these gains may have been accompanied by leakage to nontargeted areas (24, 73). In our context, companies typically adopted ZDCs covering all of their operations, whether in Indonesia or abroad. As a result, direct leakage from their demand is likely low. Furthermore, since we find little evidence of additionality, it is unlikely that ZDC adoption has affected palm oil markets in a way that would encourage leakage to non-ZDC buyers. Combining supply chain exclusion mechanisms like ZDCs with collaborative landscape approaches between companies and local government officials (74, 75) may help to overcome both leakage and additionality concerns, at least at local to regional scales.

Importantly, we emphasize that our result of no additionality should not lead companies in the oil palm sector to abandon their commitments, or civil society organizations to stop pressuring companies to eliminate deforestation from their supply chains. Our findings must be considered within the broader context of simultaneous ZDC implementation and recent deforestation reductions in Indonesia which may be driven by interactions between improved public forest governance and low economic incentives. If these policy and economic conditions change, as they did in Brazil to drive increases in deforestation circa 2015 after a decade of decline in forest loss, ZDCs may prove a critical safeguard for Indonesia’s remaining carbon-rich and biodiverse forests.

## 3. Materials and Methods

### 3.1. Data Description.

We integrated several data sources to understand the coverage and impact of company-level zero-deforestation commitments on deforestation. These sources include data describing: a) oil palm concession boundaries, b) palm oil mill locations, c) forest land cover, d) industrial oil palm plantation land cover, e) ownership and sourcing links between mills and “companies” (i.e. either mill-owning or refining company groups), f) sourcing links between mills and concessions, and g) companies’ ZDC quality.

The oil palm concession boundaries dataset a) was assembled from several sources including manually digitized concessions from the Roundtable on Sustainable Palm Oil (RSPO) audits and reports, Indonesian government data, NGO reports, and data provided by palm oil companies (see details in ref. 76). The data were cleaned, merged, and then duplicates and overlaps between concessions were removed following ref. 76.

Palm oil mill locations b) were identified using a comprehensive database of all palm oil mills in operation in Indonesia in 2022 (77). Supply-chain linkages from mills to mill-owning or refining company groups e) were defined using data from Trase available for 2018–2020 (78). These data are designed to describe trading relationships that are consistent with public traceability reports released by Indonesia’s primary palm oil mill-owning, refining, and exporting company groups. The dataset f) on sourcing relationships between mills and upstream concessions was based on data from Trase, which seeks to link mills to concessions based on RSPO declarations and the names and locations of the concessions and mills (see ref. 78, page 18). It is worth cautioning that this results in a conservative linking, with many concessions not linked to any mill. This dataset was supplemented with data from ref. 72 to obtain the median number of distinct industrial plantations per year per mill reported in the text. Ref. 72 is based on a random sample of 88 RSPO-certified mills in Indonesia; using this sample, we quantified the number of uniquely named industrial, smallholder, and unknown suppliers from which these mills sourced in each year with available data between 2009 and 2022.

We compiled comprehensive data about ZDC commitment quality g) by computing scores for 51 company groups that own or source from Indonesian palm oil mills–48 sourced from the Sustainable Palm Oil Transparency Toolkit (SPOTT) database (79) and 3 through manual assessments using the same SPOTT scoring method. We selected 15 indicators from the SPOTT database to evaluate companies’ ZDC policy design and implementation, see *SI Appendix*, Table S21. Each indicator was evaluated according to a binary ZDC quality score, representing the presence or absence of a zero-deforestation commitment. Only companies committed to zero deforestation across all their suppliers were assigned a ZDC score, while companies without such a commitment were assigned a *zero* score, see ref. 68 for full details. In a robustness test, we distinguish between low- and high-quality ZDCs, depending on whether all indicators met the required criteria (*high* score) or only some indicators did (*low* score).

**Fig. 5.**
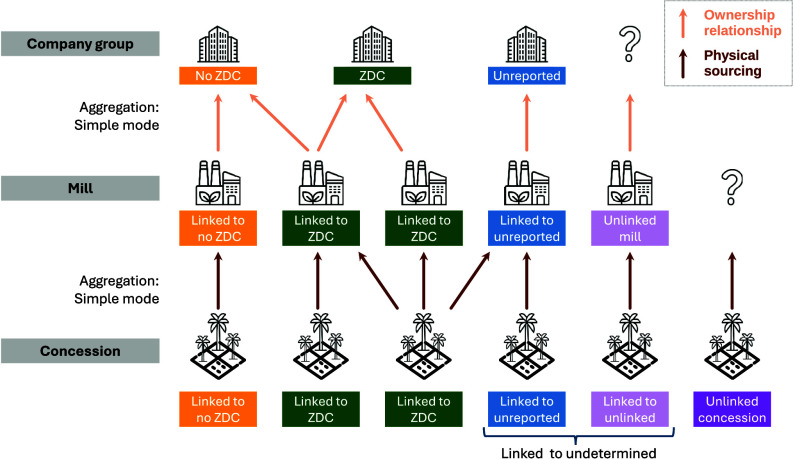
Illustration of the owner-based attribution.

Having data on ZDC quality at the company level (either mill-owning or refining company groups) and supply-chain data linking companies to mills and then to concessions, we assigned a ZDC score to each concession. To do so, we first assigned each mill a score based on their linkages to mill-owning or refining company groups, and then assigned each concession a score based on their linkages to the mills. The ZDC score attribution required addressing two challenges: uncertainty (and its propagation) and aggregation, both illustrated in Fig. 5 and *SI Appendix*, Fig. S9. The uncertainty challenge arose from two sources. First, information could be missing at different levels of the supply chain: absence of public reports at the company level (*unreported*), mills missing linkage information to companies (*unlinked*), or concessions missing linkage information to mills (*unlinked*). Second, uncertainty could propagate through existing linkages when a downstream entity lacked information. In this case, mills could be linked to companies without reports (*linked to unreported*), and concessions could be linked either to unlinked mills (*linked to unlinked*) or to mills linked to unreported companies (*linked to unreported*). Given their small numbers, concessions *linked to unlinked* and *linked to unreported* were grouped into a single category, *linked to undetermined*. While companies without public commitment reports likely did not have a ZDC, we conservatively considered them as *Unreported* instead of *No ZDC* to reflect the uncertainty, and its propagation, inherent to such an attribution exercise.

The aggregation challenge stemmed from the fact that a concession can be linked to multiple mills, and a mill to multiple companies, requiring explicit rules to derive a single ZDC score. This raised the question of how to aggregate multiple ZDC quality scores into one (either from companies to mill or from mills to concessions), a question that, to the best of our knowledge, has not been addressed in the literature so far. We addressed this by using a mode-based aggregation rule, assigning to each upstream entity the most frequent ZDC score among its linked downstream entities. We did this separately for mill-owning and refining company groups, resulting in what we call the *ZDC owner attribution* and *ZDC buyer attribution*, or, equivalently, the *ownership* and *sourcing* linkages. The aggregation procedure differed across supply-chain levels due to differences in data availability. Trase provides annual trade volumes for company–mill links, whereas mill–concession links lack volumetric information and are not time-varying. As a result, for the companies-to-mill attribution step, we included the trade volume information by using a weighted mode for each year, and aggregated annual scores into a final mill score using a simple mode. For the mills-to-concession step, we used a single unweighted mode aggregation, since we had neither volume nor year-specific information.

Deforestation data c) for each concession were computed using the Global Forest Change dataset (80), setting a threshold of 90% tree canopy cover to define pixels as initial forest in 2000. We used (81) and (13) to exclude pixels that had already been planted with timber, rubber, mixed crop plantations, and oil palm in 2000. We then used (13) to distinguish deforestation linked to industrial plantations from smallholder plantations or nonpalm plantations. We used deforestation from industrial oil palm expansion as the primary outcome in the analysis and total deforestation driven by industrial, smallholder, and nonpalm expansion as a robustness test in *SI Appendix*, Table S10. Ideally, one would consider only deforestation occurring on peatland or forest defined according to the High Carbon Stock (HCS) and High Conservation Value (HCV) frameworks to closely align with companies’ ZDCs. However, such data are difficult to obtain, incomplete, and not consistently updated. Consequently, our measure of deforestation should be considered a proxy that may include some deforestation not infringing on companies’ ZDCs. We construct deforestation rates as the annualized change in forest cover, that is, the change from year *t* to t+1 divided by the forest cover at year *t*.

### 3.2. Methods.

#### 3.2.1. Stickiness measures.

To compute the stickiness of supply-chain linkages, we adapt equations (1) to (4) in ref. 8 modified for our two-layer mill-company network. Let $a_{\mathrm{ij}}\left(t\right)$ be a 0/1 variable indicating whether there is a link between mill *i* and company *j* at time *t*. $C_{i}\left(t,t+1\right)$, the stickiness between year *t* and t+1 for mill *i*, is defined as

[1] $$\begin{array}{c} C_{i}\left(t,t+1\right)\equiv \frac{\sum_{j}a_{\mathrm{ij}}\left(t\right)a_{\mathrm{ij}}\left(t+1\right)}{\sqrt{\left[\sum_{j}a_{\mathrm{ij}}\left(t\right)\right]\left[\sum_{j}a_{\mathrm{ij}}\left(t+1\right)\right]}} \end{array}$$

When a unit has no links at time *t* or t+1, this quantity is undefined, and we set it to 0 if unit *i* was at least connected once to any unit *j*, or discard it if it was fully disconnected at both times *t* and t+1. The unit-level and time-specific stickiness $C_{i}\left(t,t+1\right)$ is then aggregated over time and over units following equations (2) to (4) in ref. 8 to obtain the total mill-level stickiness coefficient. Likewise, we invert the *j* and *i* dimensions to obtain the total company-level stickiness coefficient.

#### 3.2.2. Difference-in-differences model.

To measure the additionality of ZDCs in reducing deforestation, we use a difference-in-differences (DiD) model. A DiD model compares treated and control groups before and after a policy, measuring whether the policy led to a change in the initial difference between control and treated. Our “policy” variable is the ZDC status, which is either “no ZDC” or “ZDC.” The DiD coefficient can be given a causal interpretation as the average treatment effect under the “parallel trends” assumption. This assumption states that the initial difference between treated and control units would have remained constant absent the policy.

To define the preintervention period for the DiD, we use 2012 as the last preintervention year, noting that the majority of companies announced their pledges between 2013 and 2016 (*SI Appendix*, Fig. S1), and refer to 2001–2012 as the “before ZDC implementation” period. Given the gradual nature of ZDC adoption and implementation, we divide the post-2012 period into two phases: “partial ZDC implementation” and “full ZDC implementation.” The “partial ZDC implementation” phase (2013 to 2017) marks the rollout of pledge adoption and initial implementation efforts. This phase involved establishing supplier traceability, communicating commitments to suppliers, and monitoring compliance in order to take action against noncompliant suppliers. By 2018, the start of the “full ZDC implementation,” pledges had been fully operationalized for nearly all companies (14, 44). According to ref. 82, the average target year for identified companies to achieve traceability to the mill was the end of 2017. Additionally, data collected by Palmoil.io also shows a sharp increase in the use of palm oil company grievance trackers during the 2018–2020 period compared to 2010–2017, see *SI Appendix*, Fig. S8. Taken together, these elements suggest that by 2018, critical implementation milestones–including the establishment of monitoring systems, public disclosure of related information, and traceability efforts–had largely been achieved. During this period, in response to pressure by ZDC companies and other stakeholders, the Roundtable on Sustainable Palm Oil (RSPO) also included a zero-deforestation requirement and the use of the High Carbon Stock Approach into its Principles and Criteria (83). The amendment was adopted in November 2018 and was immediately applicable for new RSPO member growers, while existing RSPO grower members were given a transition period of one year to align their operations with the new requirements (84). Nevertheless, since many companies have chosen to implement their ZDCs through RSPO certification, there is a high correlation between ZDC adoption and RSPO certification. Given this coupled implementation, and the challenges of disentangling causal effects, we interpret our treatment effect estimates as the aggregate impact of ZDCs, including their use of RSPO as an implementation mechanism.

In our main DiD analysis, we focus on the “full ZDC implementation” period as the (post) intervention period, while treating the “partial ZDC implementation” period as part of the (post) intervention period in robustness tests (*SI Appendix*, Table S6). This modeling choice reflects the limited implementation observed between 2013 and 2017, as well as the availability of Trase volumetric supply chain data only for the 2018–2020 period.

Our unit of analysis is an oil palm concession, with industrial-palm-driven deforestation as the primary outcome of interest. By focusing on concessions that could be clearly linked to mills, we target the areas where palm oil companies have the most direct influence in implementing their policies. A key limitation of this approach is that it assesses compliance and additionality within the supply chain but not at the broader landscape level. Expanding the analysis beyond concessions, though methodologically different, is a crucial direction for future research, as evidenced by ref. 24 regarding the RSPO mandate. We estimate our DiD model on a subset of the concessions that met a set of criteria such as a positive level of forest cover in 2000 and the presence of industrial-palm-driven deforestation. See *SI Appendix*, section C.1 and Table S19 for details on the concession selection criteria as well as *SI Appendix*, Tables S4 and S12 for robustness tests assessing the sensitivity of our results to these selection criteria.

Our outcome of interest is industrial-palm-driven deforestation, obtained by merging (80) with (13). This focus is motivated by both the research question and data constraints. This type of deforestation is primarily controlled by large-scale palm oil corporations, making it the most relevant target for our analysis. Additionally, smallholder-palm-driven deforestation accounts for only 5% of total deforestation within the concession dataset and is predicted with lower accuracy by ref. 13.

The DiD is estimated using the following two-way fixed-effect panel model:

[2] $$\begin{array}{c} y_{\mathrm{it}}=\alpha_{i}+\alpha_{t}+\beta D_{\mathrm{it}}^{K}+\varepsilon_{\mathrm{it}}, \end{array}$$

where the index *i* refers to the unit (concessions) while the index *t* corresponds to the time period. $\alpha_{i}$ and $\alpha_{t}$ are respectively the unit and time fixed effects and $y_{\mathrm{it}}$ is the deforestation/forest cover variable of interest. $D_{\mathrm{it}}^{K}$, with K∈{owner,buyer}, is a dummy variable taking a value of 0 during 2001–2012 and a value of 1 in 2018–2020 for the units that have a “ZDC” status and 0 for those that have a “no ZDC” status, where the definition of “ZDC” is based either on the owner or sourcing (buyer) attribution.

Because we observe supply-chain linkages only in 2018–2020, we cannot observe whether a concession classified as “linked to ZDC” during the *post* period was also “linked to ZDC” during the *pre* period, and vice-versa. Consequently, our DiD approach uses as treated units those concessions that were either *persistently linked to ZDC companies* or *newly linked to ZDC companies* (linked to ZDC companies during the *post* but not *pre* period). Likewise, our control group consists of concessions that were either *never linked to ZDC companies* or *previously linked to ZDC companies* (linked to ZDC companies during the *pre* but not *post* period). In *SI Appendix*, section B, we show that in such a setting, the DiD estimates a (weighted) combination of three unobservable DiD estimators: the DiD on the *persistently-linked-to-ZDC*, the DiD on the *newly-linked-to-ZDC*, and the DiD on the *previously-linked-to-ZDC*, all evaluated using the *never-linked-to-ZDC* as control group. Assuming that each of these unobservable subgroups follows parallel trends with respect to the *never-linked-to-ZDC* subgroup in the absence of treatment, we show that our DiD identifies a convex combination of the average treatment effect on the treated (ATT) on the *persistently-linked-to-ZDC* and the *newly-linked-to-ZDC* concessions. Furthermore, we demonstrate that under a relaxation of the parallel trends assumption, wherein ZDC companies maintain compliant concessions while excluding concessions with deforestation, our DiD estimator yields an upper bound on the weighted combination of ATTs. See *SI Appendix*, section B for full details.

A further complication arises due to the fact that we do not observe treatment status during the *partial ZDC implementation* period. If, during that period, some concessions effectively reduce their deforestation under ZDC but are no longer linked to ZDC companies during the *full ZDC implementation* period, this could introduce underestimation bias. The intuition behind this is that partially treated units would act as control units; for a formal discussion see *SI Appendix*, section B. Such a phenomenon is more likely to happen with the sourcing-based ZDC attribution, given the low stickiness of the linkages, so that the DiD estimates based on sourcing linkages should be interpreted with caution. As a robustness check, we restrict the sample to concessions that were permanently linked to ZDC companies versus those that were never linked to ZDC, and obtain qualitatively similar estimates (*SI Appendix*,Table S13).

To examine whether the parallel trends assumption is likely to hold, we run a parallel pretrends test. This is obtained by running an event study, normalizing the coefficient at time −1 (2011) to be 0 (*SI Appendix*, Fig. S5), and then running a Wald test for the joint hypothesis that all normalized event-study coefficients before the intervention are 0. A low *P*-value would indicate that the null hypothesis of parallel trends is rejected, casting doubt on the interpretation of the DiD coefficient as causal.

In some robustness checks, we further disaggregated the ZDC score into “High” and “Low” (*Data Description*). This allowed us to run three DiD models: the first two comparing either “High ZDC” or “Low ZDC” to the control group of “No ZDC,” the last one comparing “High ZDC” to the control group of “Low ZDC;” see *SI Appendix*, Table S7.

## Supplementary Material

Appendix 01 (PDF)

## Data Availability

Code and datasets to replicate the figures and tables of the paper and appendix have been deposited on Zenodo (85).

## Footnotes

^*^A notable exception is (38) who were able to analyze nearly all treated units across large areas in Brazil due to the near-universal adoption of the soy ZDC within its targeted region, the Amazon biome.

^†^It should be noted, however, that some of the detected deforestation—such as clearance of community-managed agroforests—might occur on land that is neither peatland nor High Carbon Stock Approach/High Conservation Value and thus fall outside the scope of ZDCs (*Data Description*).

^‡^Formally, both coefficients could be nonsignificant yet still significantly different from each other. We tested this and found that we cannot reject the null hypothesis that both coefficients are equal.
