# Supplementary material for: Zero-deforestation commitments in Indonesia’s palm oil sector achieve high compliance but no additionality
Source: Proc Natl Acad Sci U S A. 2026 Jul 13;123(29):e2511503123. doi: 10.1073/pnas.2511503123 (PMC13389643; doi:10.1073/pnas.2511503123)
Supplement: Supplementary file 1 — Appendix 01 (PDF) [file pnas.2511503123.sapp.pdf]

Supporting Information for: *Zero-deforestation commitments in Indonesia's palm oil sector achieve high compliance but no additionality*

Matthieu Stigler, Janina Grabs, Robert Heilmayr, Kimberly Carlson, Adelina Chandra, Jason Jon Benedict, Rachael Garrett

May 6, 2026

Contents

SI A Supplementary tables and figures . . . 1
SI B Formalization of the DiD approach . . . 14
SI C Supplementary details on data assembly . . . 17
SI C.1 Data selection rules . . . 17
SI C.2 Clustering procedure . . . 18

SI A Supplementary tables and figures

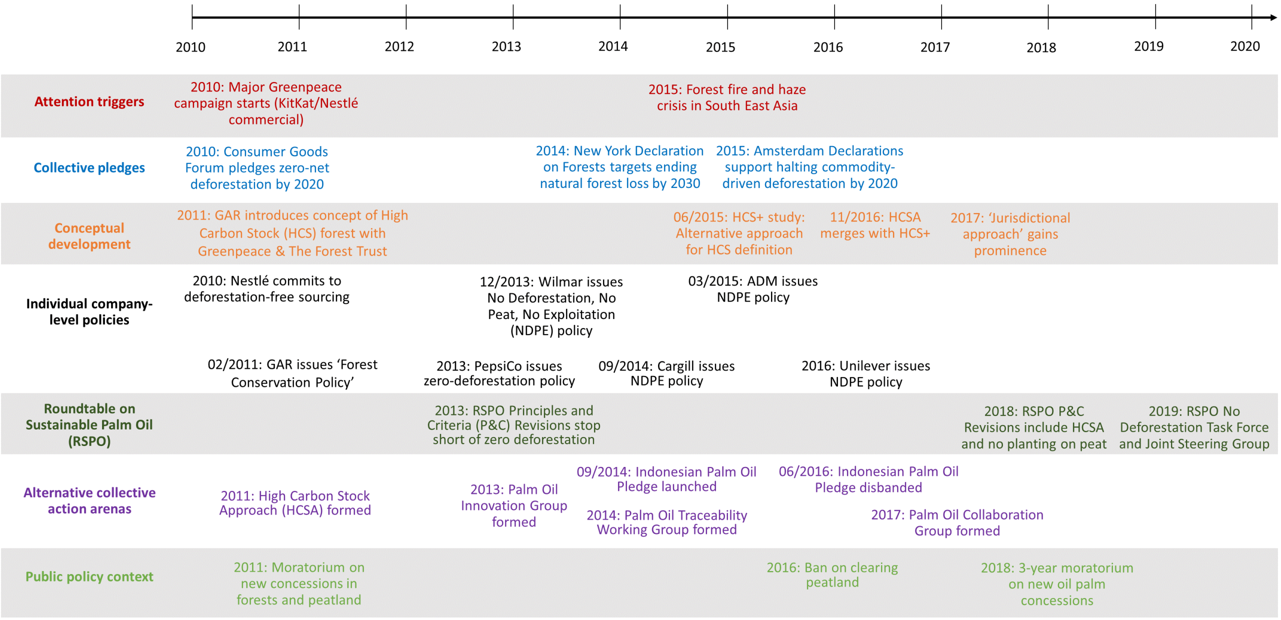

Figure SI.F1: Timeline of ZDC implementation steps

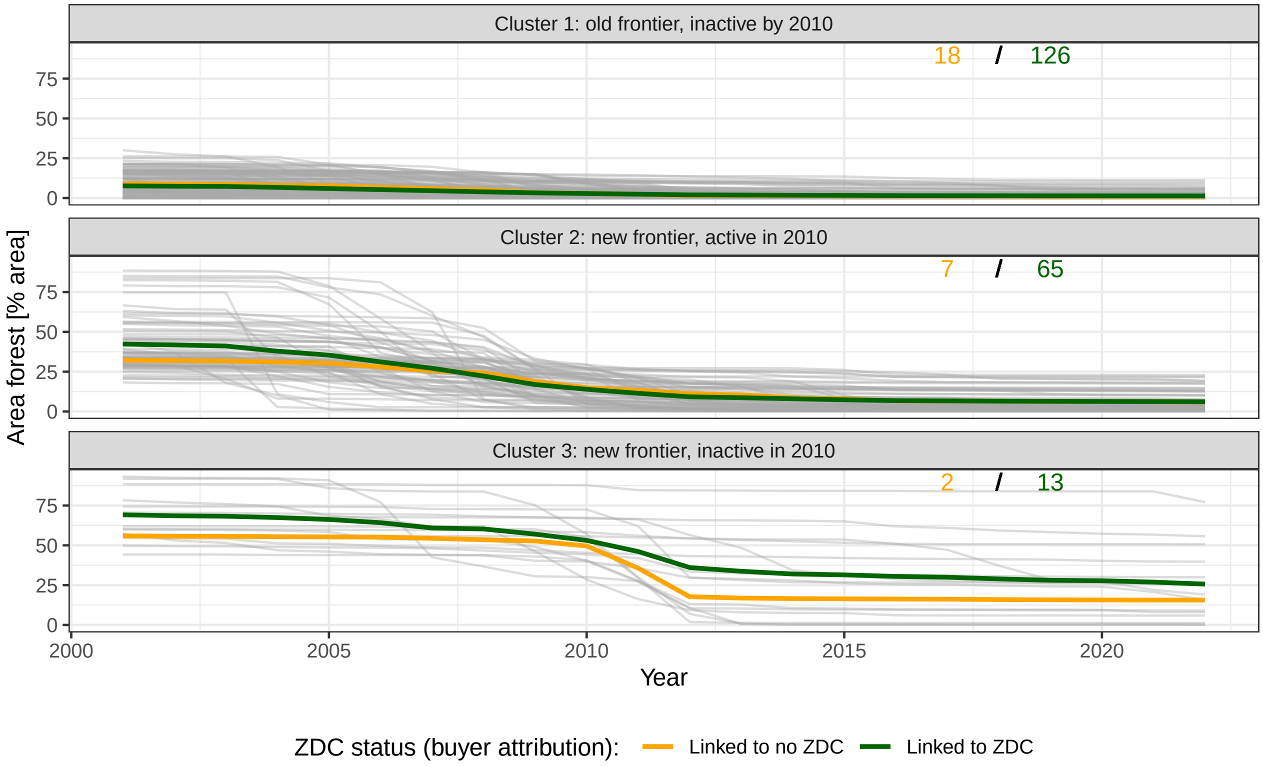

Figure SI.F2: Forest cover for each concession, together with the average across ZDC and non-ZDC groups, buyer-based score.

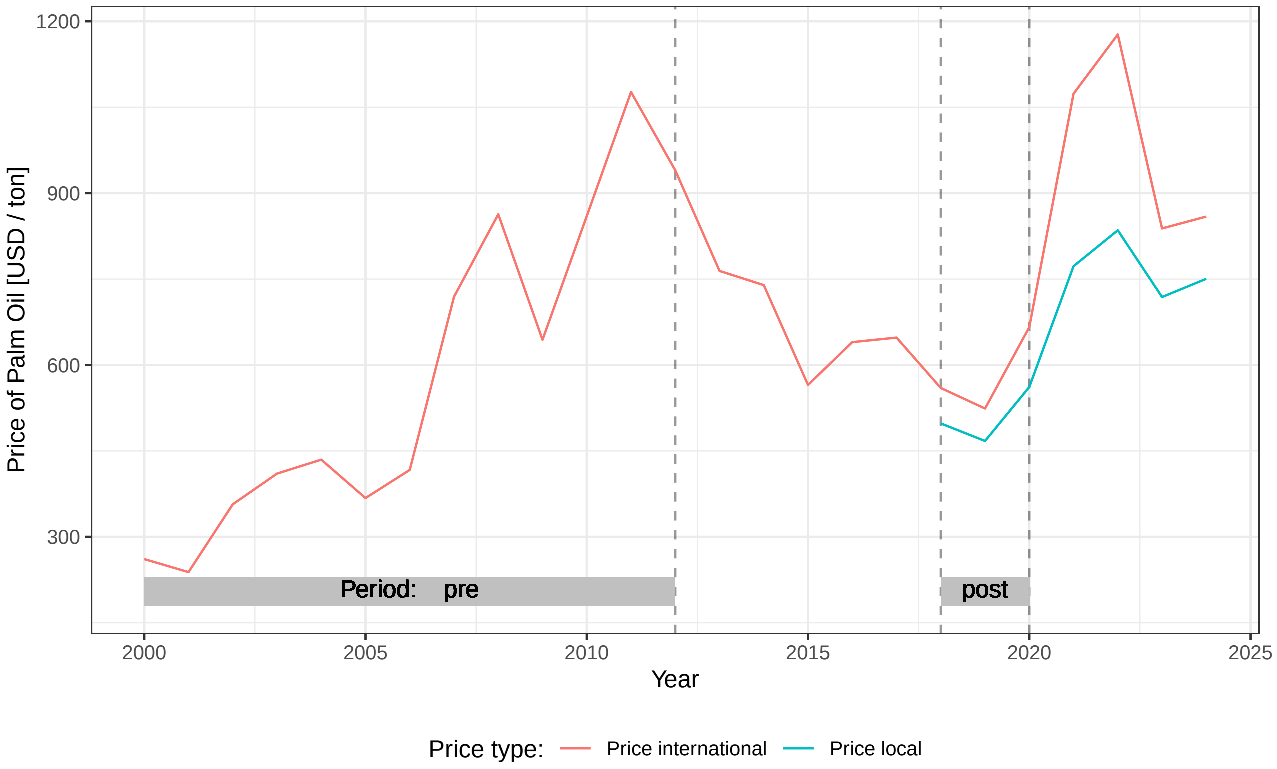

Figure SI.F3: Evolution of palm oil prices. The *international price* corresponds to the annual average of the Malaysia Palm Oil Futures (first contract forward), obtained from the International Monetary Fund and accessed through the FRED database of the Federal Reserve Bank of St. Louis (series code [PPOILUSDM]; https://fred.stlouisfed.org/series/PPOILUSDM, retrieved on May 21, 2024). The *local price* corresponds to the annual average of province-level prices from the Ministry of Agriculture's Rekapitulasi Harga TBS 2025 (https://12ap.pertanian.go.id/sipasbun2020/tbs.php, retrieved in November 2025).

Table SI.T1: Descriptive statistics of the sample, by ZDC score

Variable	Unlinked	Linked to Undetermined	Linked to no ZDC	Linked to low ZDC	Linked to high ZDC	Linked to ZDC (low+high)
	ZDC status					
	ZDC owner attribution					
N initial sample	1620	345	234	209	236	445
N remove: not in main island	145	1				
N remove: 0 forest 2000	122	22	14	7	22	29
N remove: 0 industrial-palm deforestation	1034	93	70	48	69	117
N final sample	584	252	164	161	167	328
Average distance to first mill [km]	14.66 (0.58)	7.05 (0.33)	8.87 (0.73)	7.70 (0.59)	7.64 (0.47)	7.67 (0.37)
Average concession area [km^2]	85.38 (3.67)	90.44 (4.7)	91.79 (5.28)	109.83 (6.87)	121.47 (6.23)	115.76 (4.63)
Oil palm suitability index (%)	6333.99 (59.23)	6333.58 (85.41)	6359.92 (100.94)	6519.06 (97.94)	6497.17 (89.23)	6507.92 (66.04)
Average forest cover in 2000	38.31 (2.44)	31.65 (2.77)	32.24 (3.5)	29.25 (3.3)	35.10 (3.66)	32.23 (2.47)
Average forest cover in 2012	24.07 (1.06)	12.08 (1.13)	11.11 (1.27)	10.87 (1.29)	11.29 (1.49)	11.08 (0.99)
Average deforestation (annualized)	3.36 (0.15)	5.66 (0.26)	5.33 (0.33)	4.94 (0.32)	5.24 (0.35)	5.09 (0.23)
Average deforestation 2001-12 (annualized)	4.05 (0.23)	7.58 (0.4)	7.12 (0.53)	6.72 (0.48)	7.46 (0.55)	7.10 (0.36)
Average deforestation 2018-20 (annualized)	1.28 (0.16)	1.16 (0.29)	0.62 (0.15)	0.51 (0.12)	0.44 (0.1)	0.48 (0.08)
	ZDC buyer attribution					
N initial sample	1620	662	32	94	236	330
N remove: not in main island	145	1				
N remove: 0 forest 2000	122	26		2	37	39
N remove: 0 industrial-palm deforestation	1034	149	5	15	111	126
N final sample	584	513	27	79	125	204
Average distance to first mill [km]	14.66 (0.58)	8.24 (0.33)	7.19 (0.98)	5.73 (0.4)	6.98 (0.57)	6.50 (0.38)
Average concession area [km^2]	85.38 (3.67)	102.18 (3.69)	75.65 (7.52)	91.57 (6.13)	112.94 (6.55)	104.66 (4.71)
Oil palm suitability index (%)	6333.99 (59.23)	6286.04 (58.38)	7003.21 (210.44)	6594.50 (119.96)	6711.15 (101.63)	6665.98 (77.61)
Average forest cover in 2000	38.31 (2.44)	36.07 (2.18)	15.07 (2.92)	26.36 (2.85)	22.72 (2.88)	24.13 (2.08)
Average forest cover in 2012	24.07 (1.06)	13.72 (0.85)	5.35 (1.6)	9.52 (1.64)	4.55 (0.77)	6.47 (0.81)
Average deforestation (annualized)	3.36 (0.15)	5.10 (0.18)	6.73 (0.65)	5.63 (0.5)	5.82 (0.42)	5.75 (0.32)
Average deforestation 2001-12 (annualized)	4.05 (0.23)	6.69 (0.27)	9.56 (1.09)	8.28 (0.78)	8.51 (0.66)	8.42 (0.5)
Average deforestation 2018-20 (annualized)	1.28 (0.16)	0.85 (0.15)	1.06 (0.58)	0.43 (0.15)	0.42 (0.1)	0.42 (0.08)

The table gives various counts or averages, together with standard errors for the averages. Results are disaggregated by ZDC score.

Table SI.T2: DiD results for the benchmark specification

	ZDC owner attribution	ZDC buyer attribution
ZDC treatment	−0.12	0.49
	(0.67)	(1.44)
Num. obs.	7380	3465
Num. control:	164	27
Num. treated:	328	204
Parallel test: Wald stat	10.40	41.43
Parallel test: p-val	0.49	0.00

$^{***}p < 0.001$; $^{**}p < 0.01$; $^{*}p < 0.05$. Standard errors clustered at the concession level.

Table SI.T3: DiD results by frontier-stage cluster

	ZDC owner attribution				ZDC buyer attribution			
	All	Cluster: 1	Cluster: 2	Cluster: 3	All	Cluster: 1	Cluster: 2	Cluster: 3
ZDC treatment	−0.12	−0.78	0.88	0.98	0.49	2.22	-3.75^{*}	1.91
	(0.67)	(0.89)	(1.18)	(1.03)	(1.44)	(1.92)	(1.85)	(4.46)
Num. obs.	7380	4380	2250	750	3465	2160	1080	225
Num. control:	164	91	53	20	27	18	7	2
Num. treated:	328	201	97	30	204	126	65	13
Parallel test: Wald stat	10.40	10.63	8.05	8.61	41.43	43.02	46.66	46913.90
Parallel test: p-val	0.49	0.47	0.71	0.66	0.00	0.00	0.00	0.00

$^{***}p < 0.001$; $^{**}p < 0.01$; $^{*}p < 0.05$. Standard errors clustered at the concession level.
Column *All* refers to the benchmark regression including all clusters, and therefore corresponds to Table 1 in the paper.
Columns with *Cluster* refer to the regressions run for each frontier-stage cluster separately. We interpret the clusters as respectively: "Old frontier, inactive by 2010", "New frontier, active in 2010", and "New frontier, inactive in 2010" see SI C.2 for details on how the clusters are constructed and interpreted.

Table SI.T4: DiD results using alternative categories as control group

	ZDC owner attribution			ZDC buyer attribution		
Control group:	Unlinked	Linked to Undetermined	Linked to no ZDC	Unlinked	Linked to Undetermined	Linked to no ZDC
ZDC treatment	-3.85^{***}	−0.20	−0.12	-5.23^{***}	-2.16^{***}	0.49
	(0.47)	(0.64)	(0.67)	(0.58)	(0.61)	(1.44)
Num. obs.	13680	8700	7380	11820	10755	3465
Num. control:	584	252	164	584	513	27
Num. treated:	328	328	328	204	204	204
Parallel test: Wald stat	47.41	12.89	10.40	54.52	18.60	41.43
Parallel test: p-val	0.00	0.30	0.49	0.00	0.07	0.00

$^{***}p < 0.001$; $^{**}p < 0.01$; $^{*}p < 0.05$. Standard errors clustered at the concession level.
Outcome variable: Deforested area [% annualized]

Table SI.T5: DiD results within each island

	Kalimantan		Papua	Sulawesi	Sumatra	
ZDC attribution:	Owner	Buyer	Owner	Owner	Owner	Buyer
ZDC treatment	0.36	−0.25	1.55	0.51	−1.79	3.32
	(0.82)	(1.62)	(1.25)	(1.30)	(1.29)	(1.94)
Num. obs.	4740	2115	300	90	2250	1320
Num. control:	114	24	8	1	41	3
Num. treated:	202	117	12	5	109	85
Parallel test: Wald stat	11.09	31.23	23.90	1445.92	6.80	269.99
Parallel test: p-val	0.44	0.00	0.01	0.00	0.82	0.00

$^{***}p < 0.001$; $^{**}p < 0.01$; $^{*}p < 0.05$. Standard errors clustered at the concession level.
Outcome variable: Deforested area [% annualized].
Depending on the ZDC attribution method, some islands do not have enough units to run a difference-in-differences model.

Table SI.T6: DiD results changing the pre/post periods

	01-12 vs 18-20		01-12 vs 13-20		01-10 vs 18-20		01-12 vs 18-22		01-12 vs 13-17	
ZDC attribution:	Owner	Buyer	Owner	Buyer	Owner	Buyer	Owner	Buyer	Owner	Buyer
ZDC treatment	−0.12	0.49	−0.53	0.37	−0.68	−1.04	0.02	0.84	−0.77	0.30
	(0.67)	(1.44)	(0.76)	(1.48)	(0.66)	(1.44)	(0.65)	(1.31)	(0.89)	(1.68)
Num. obs.	7380	3465	9840	4620	6396	3003	8364	3927	8364	3927
Num. control:	164	27	164	27	164	27	164	27	164	27
Num. treated:	328	204	328	204	328	204	328	204	328	204
Num. periods:	15	15	20	20	13	13	17	17	17	17
Parallel test: Wald stat	10.40	41.43	10.40	41.42	6.16	36.72	10.40	41.43	10.40	41.43
Parallel test: p-val	0.49	0.00	0.49	0.00	0.72	0.00	0.49	0.00	0.49	0.00

$^{***}p < 0.001$; $^{**}p < 0.01$; $^{*}p < 0.05$. Standard errors clustered at the concession level.
Outcome variable: Deforested area [% annualized]

Table SI.T7: DiD results with disaggregated ZDC score

	ZDC owner attribution				ZDC buyer attribution			
Control group:	No ZDC	No ZDC	ZDC low	No ZDC	No ZDC	No ZDC	ZDC low	No ZDC
Treat: ZDC low	0.29				0.65			
	(0.75)				(1.57)			
Treat: ZDC high		−0.53	−0.82			0.40	−0.25	
		(0.79)	(0.76)			(1.50)	(1.05)	
Treat: ZDC low+high				−0.12				0.49
				(0.67)				(1.44)
Num. obs.	4875	4965	4920	7380	1590	2280	3060	3465
Num. control:	164	164	161	164	27	27	79	27
Num. treated:	161	167	167	328	79	125	125	204
Parallel test: Wald stat	8.17	15.35	12.77	10.40	21.05	38.33	7.73	41.43
Parallel test: p-val	0.70	0.17	0.31	0.49	0.03	0.00	0.74	0.00

$^{***}p < 0.001$; $^{**}p < 0.01$; $^{*}p < 0.05$. Standard errors clustered at the concession level. Outcome variable: Deforested area [% annualized].
The rows starting with *Treat* indicate which treated group is considered, either *Low* ZDC, *High* ZDC, or both (*Low-High*). The columns indicate which control group is used.

Table SI.T8: DiD results using alternative definitions of deforestation

	Deforested area [hec]		Deforested area [% annualized]		Deforested area [% forest]		Deforested area [% area]	
ZDC attribution:	Owner	Buyer	Owner	Buyer	Owner	Buyer	Owner	Buyer
ZDC treatment	0.28	-0.58^{**}	−0.12	0.49	0.03	0.51	0.36^{*}	−0.27
	(0.20)	(0.18)	(0.67)	(1.44)	(0.28)	(0.62)	(0.16)	(0.24)
Num. obs.	7380	3465	7380	3465	7380	3465	7380	3465
Num. control:	164	27	164	27	164	27	164	27
Num. treated:	328	204	328	204	328	204	328	204
Parallel test: Wald stat	6.10	35.05	10.40	41.43	8.88	45.88	11.07	23.45
Parallel test: p-val	0.87	0.00	0.49	0.00	0.63	0.00	0.44	0.02

$^{***}p < 0.001$; $^{**}p < 0.01$; $^{*}p < 0.05$

Deforested area [hectare]: year-to-year deforestation
Deforested area [% annualized]: year-to-year deforestation divided by the concession's forest area the preceding year
Deforested area [% forest]: the year-to-year deforestation divided by the concession's forest area in 2000
Deforested area [% area]: the year-to-year deforestation divided by the concession's area

Table SI.T9: DiD results using forest area instead of deforestation

	Area forest [hec]		Area forest [% area]	
ZDC attribution:	Owner	Buyer	Owner	Buyer
ZDC treatment	1.96	-3.84^{*}	3.02^{*}	-0.56
	(2.13)	(1.77)	(1.51)	(2.35)
Num. obs.	7380	3465	7380	3465
Num. control:	164	27	164	27
Num. treated:	328	204	328	204
Parallel test: Wald stat	5.64	31.15	7.20	25.85
Parallel test: p-val	0.90	0.00	0.78	0.01

$^{***}p < 0.001$; $^{**}p < 0.01$; $^{*}p < 0.05$. Standard errors clustered at the concession level.
Area forest [hec]: Forest area (in hectares) in each concession.
Area forest [% area]: Forest area divided by concession's total area.

Table SI.T10: DiD results comparing industrial-palm driven and any deforestation

	ZDC owner attribution		ZDC buyer attribution	
	Any deforestation	Industrial palm deforestation	Any deforestation	Industrial palm deforestation
ZDC treatment	-0.87	-0.12	-0.64	0.49
	(0.78)	(0.67)	(1.55)	(1.44)
Num. obs.	7380	7380	3465	3465
Num. control:	164	164	27	27
Num. treated:	328	328	204	204
Parallel test: Wald stat	12.02	10.40	47.87	41.43
Parallel test: p-val	0.36	0.49	0.00	0.00

$^{***}p < 0.001$; $^{**}p < 0.01$; $^{*}p < 0.05$. Standard errors clustered at the concession level.

Table SI.T11: DiD results using the Generalized Synthetic Control and the Matrix Completion methods

	ZDC owner attribution			ZDC buyer attribution		
	Two-way FE	Generalized synthetic	Matrix Completion	Two-way FE	Generalized synthetic	Matrix Completion
ZDC treatment	-0.12	-0.38	-0.39	0.49	-0.58	0.42
	(0.67)	(0.59)	(0.52)	(1.44)	(2.13)	(1.33)
Num. obs.	7380	7380	7380	3465	3465	3465
Num. control:	164	164	164	27	27	27
Num. treated:	328	328	328	204	204	204
Hyperparameter:		2.00	0.01		1.00	0.05

$^{***}p < 0.001$; $^{**}p < 0.01$; $^{*}p < 0.05$. Standard errors clustered at the concession level.
Two-way FE refers to the standard two-way fixed effect as used above.
Generalized synthetic refers to the Generalized Synthetic Control method of Xu (1).
Matrix Completion refers to the matrix completion method of Athey et al. (2).
The row *hyperparameter* indicates either the number of factors (Generalized synthetic) or the λ penalty (matrix completion) parameter selected by cross-validation.

Table SI.T12: DiD results including concessions that never had palm-driven deforestation

	ZDC owner attribution		ZDC buyer attribution	
Control sample:	Benchmark	Include 0 palm-deforest	Benchmark	Include 0 palm-deforest
ZDC treatment	−0.12	−0.38	0.49	1.56
	(0.67)	(0.56)	(1.44)	(1.32)
Num. obs.	7380	9540	3465	4845
Num. control:	164	220	27	32
Num. treated:	328	416	204	291
Parallel test: Wald stat	10.40	10.38	41.43	38.22
Parallel test: p-val	0.49	0.50	0.00	0.00

$^{***}p < 0.001$; $^{**}p < 0.01$; $^{*}p < 0.05$. Standard errors clustered at the concession level.
Benchmark describes the standard sample used in the main text (see SI C.1) that excludes concessions that never had industrial palm-driven deforestation.
Include 0 palm-deforest includes concessions that never had industrial palm-driven deforestation.

Table SI.T13: DiD results using concessions that were persistently linked to ZDC

	ZDC owner attribution		ZDC buyer attribution	
Concessions:	All	Persistent-only	All	Persistent-only
ZDC treatment	−0.12	−0.05	0.49	−0.35
	(0.67)	(0.79)	(1.44)	(2.80)
Num. obs.	7380	5820	3465	1680
Num. control:	164	127	27	6
Num. treated:	328	261	204	106
Parallel test: Wald stat	10.40	10.82	41.43	52.41
Parallel test: p-val	0.49	0.46	0.00	0.00

$^{***}p < 0.001$; $^{**}p < 0.01$; $^{*}p < 0.05$. Standard errors clustered at the concession level.
Column *All* is the benchmark set of concessions used in the main analysis. Column *Persistent-only* uses only concessions that were persistently either ZDC or non-ZDC throughout the three years in which the linkages are observed.

Table SI.T14: DiD results using concessions that are neither linked to companies through ownership nor sourcing linkages as controls

	Benchmark		Control: neither owner- nor buyer-linked			
	Owner	Buyer	Owner and Buyer	Owner or Buyer	Owner only	Buyer only
ZDC treatment	−0.12	0.49	1.33	1.82	3.80	2.91
	(0.67)	(1.44)	(1.87)	(1.85)	(2.25)	(3.62)
Num. obs.	7380	3465	2190	2745	600	315
Num. control:	164	27	12	12	12	12
Num. treated:	328	204	134	171	28	9
Parallel test: Wald stat	10.40	41.43	69.14	74.02	27.65	14.55
Parallel test: p-val	0.49	0.00	0.00	0.00	0.00	0.20

$^{***}p < 0.001$; $^{**}p < 0.01$; $^{*}p < 0.05$. Standard errors clustered at the concession level.
Columns under *Benchmark* refer to the standard DiD using either owner- or sourcing-based ZDC scores separately.
Columns under *Control: neither owner- nor buyer-linked* refer to specifications where *control* concessions are those that are considered as control under both the owner- and sourcing-based ZDC scores. *Owner and Buyer* includes only concessions treated under both scores; *Owner or Buyer* includes those treated under at least one; and *Buyer only* and *Owner only* restrict treatment to those treated under a single score.

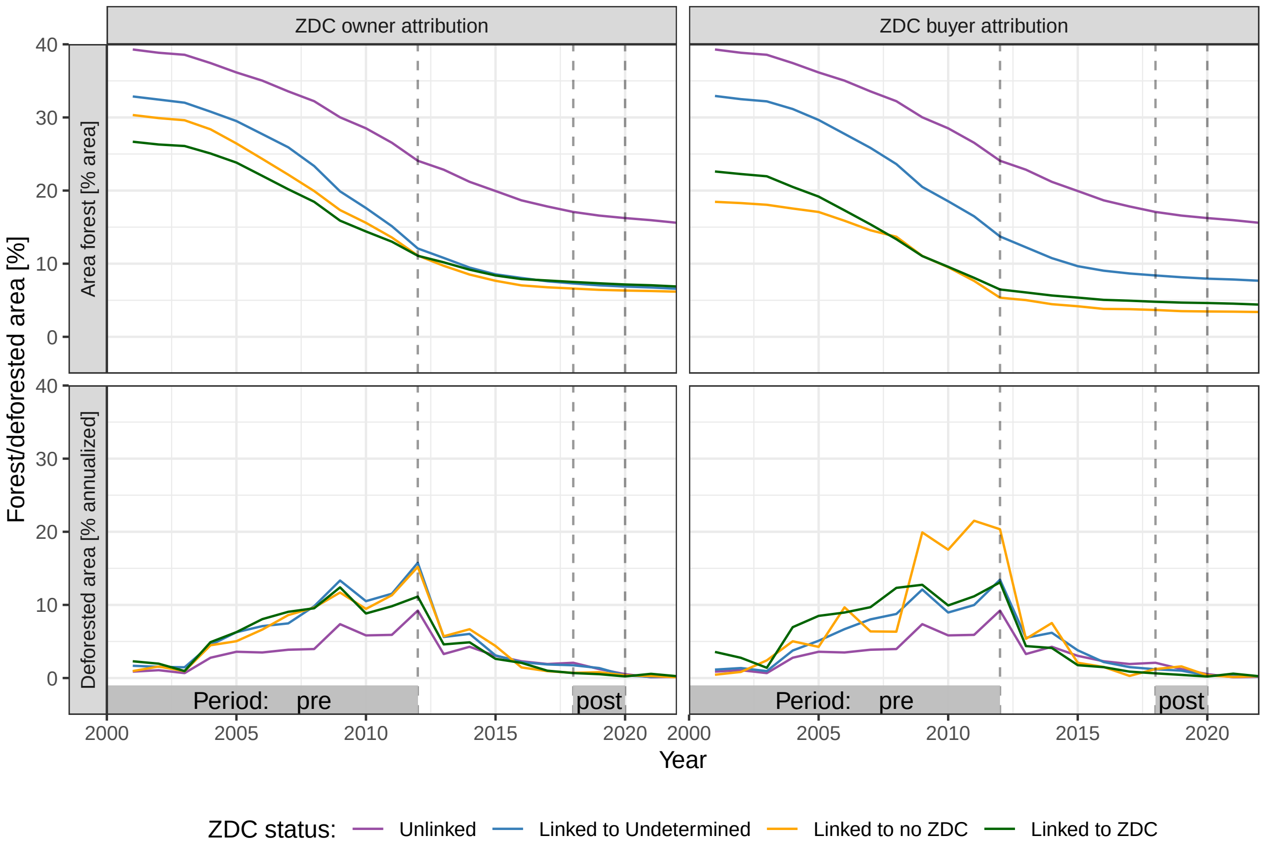

Figure SI.F4: Forest cover and deforestation rates by ZDC status, all categories

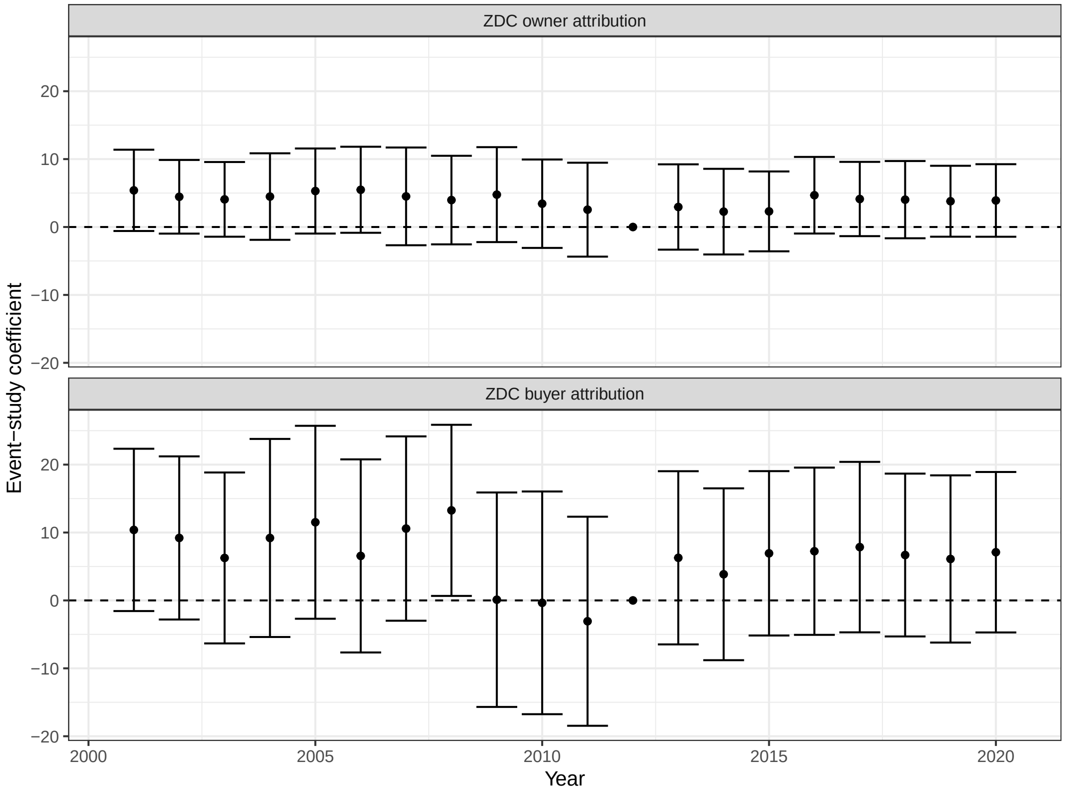

Figure SI.F5: Event study plot

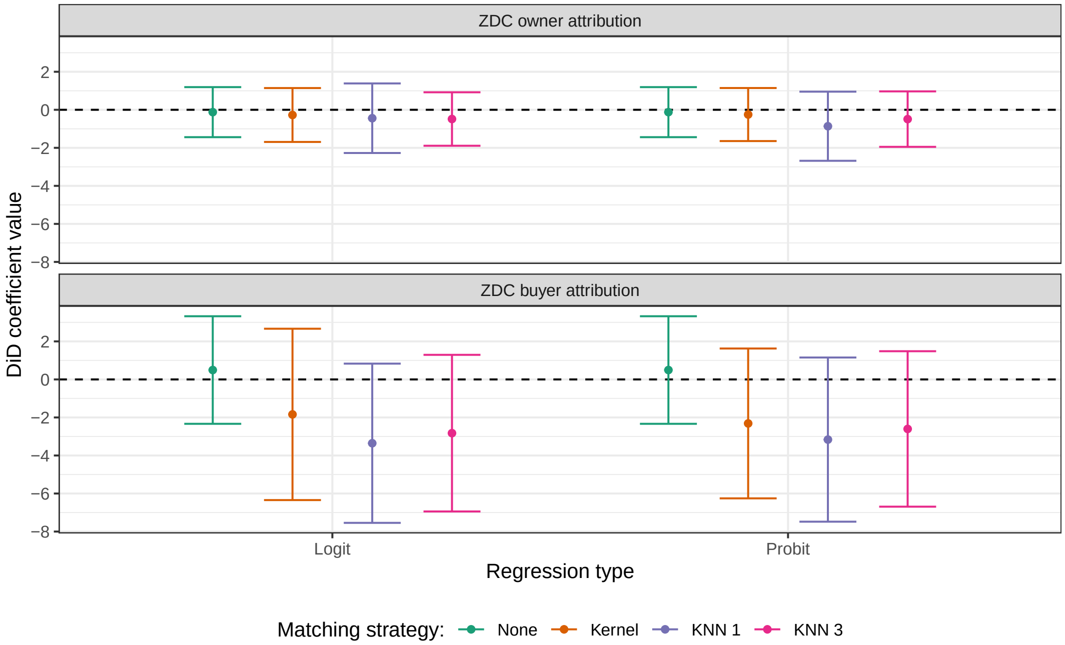

Figure SI.F6: DiD coefficients using propensity-score matching weights

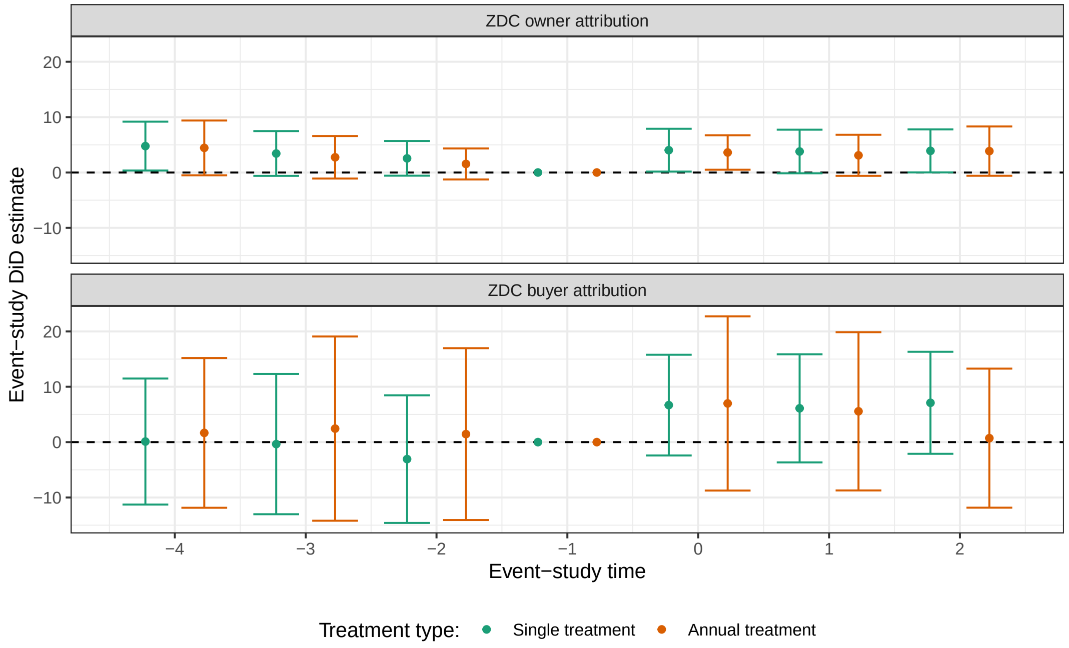

Figure SI.F7: Event study coefficients using annual treatment with the de Chaisemartin and d'Haultfeuille (2024) estimator

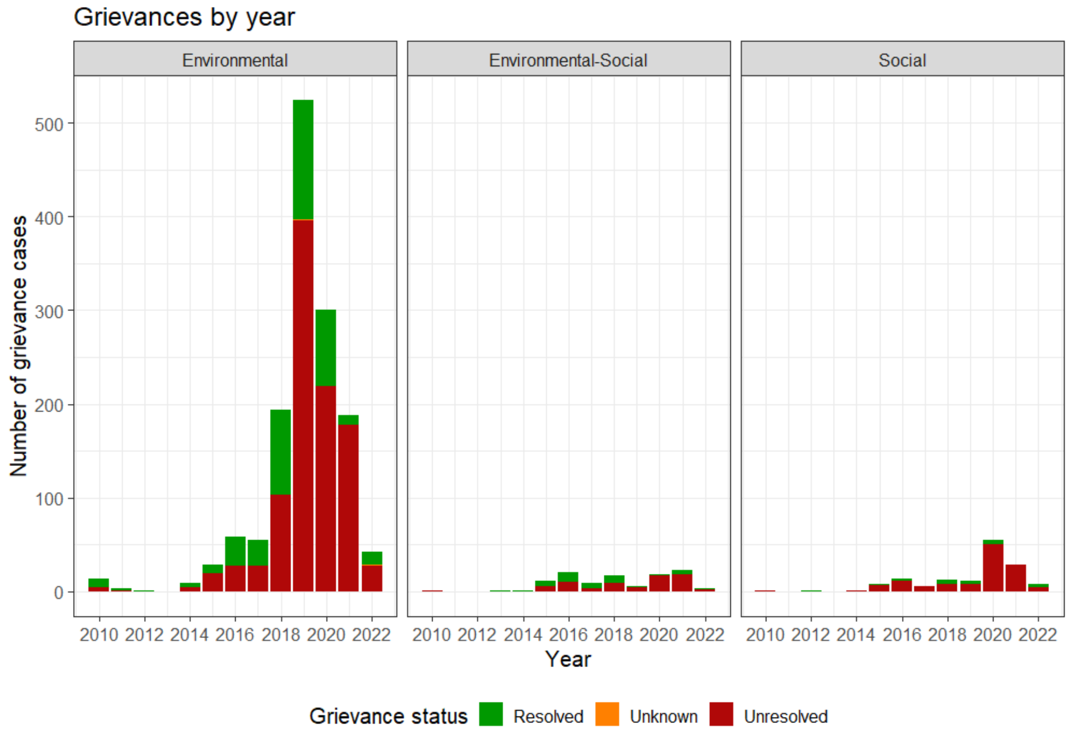

Figure SI.F8: Grievances by year. Source: own illustration using data from https://www.palmoil.io/.

Table SI.T15: Estimates of ZDC status

	ZDC owner attribution			ZDC buyer attribution		
	Linear	Probit	Logit	Linear	Probit	Logit
Distance to first mill	−0.000	−0.000	−0.000	−0.000	−0.000	−0.000
	(0.000)	(0.000)	(0.000)	(0.000)	(0.000)	(0.000)
Concession size	0.001***	0.002***	0.002***	0.000	0.001	0.001
	(0.000)	(0.000)	(0.000)	(0.000)	(0.000)	(0.000)
Concession forest % in 2000	−0.002*	−0.003**	−0.003**	0.001	0.001	0.001
	(0.001)	(0.001)	(0.001)	(0.001)	(0.001)	(0.001)
Concession forest % in 2012	0.003	0.004	0.004	−0.001	−0.002	−0.002
	(0.002)	(0.002)	(0.002)	(0.003)	(0.002)	(0.002)
Deforestation rate 2001-12	0.004	0.006	0.007	−0.013	−0.013	−0.012
	(0.009)	(0.009)	(0.009)	(0.009)	(0.009)	(0.009)
Palm oil suitability (GAEZ)	0.000	0.000	0.000	−0.000	−0.000	−0.000
	(0.000)	(0.000)	(0.000)	(0.000)	(0.000)	(0.000)
AIC	651.500	616.392	616.058	137.722	170.221	170.541
BIC	685.088	645.781	645.447	165.261	194.318	194.638
Num. obs.	492	492	492	231	231	231
Pseudo R^2	0.041	0.038	0.039	0.039	0.062	0.061
Kappa	0.012	0.051	0.070	0.000	0.000	0.000
F1	0.046	0.120	0.149			

$^{***}p < 0.001$; $^{**}p < 0.01$; $^{*}p < 0.05$

The coefficients shown are average marginal effects estimated with the R package *margins* (3). Rows (McFadden's) *Pseudo* R^2, *Kappa*, and *F1* are the goodness of fit and of classification metrics.

Table SI.T16: Matching-only strategy: propensity-score estimates

	ZDC owner attribution				ZDC buyer attribution			
Matching method:	KNN 1	KNN 3	Kernel	None	KNN 1	KNN 3	Kernel	None
Constant	0.87**	0.79***	0.82***	0.67***	2.08	1.97	2.30	1.27*
	(0.29)	(0.21)	(0.20)	(0.16)	(1.31)	(1.18)	(1.34)	(0.63)
ZDC treatment	−0.10	−0.08	−0.08	−0.05	−0.41	−0.39	−0.48	−0.21
	(0.07)	(0.06)	(0.05)	(0.04)	(0.33)	(0.30)	(0.34)	(0.16)
Num. obs.	1356	1470	1392	1476	690	693	375	693

$^{***}p < 0.001$; $^{**}p < 0.01$; $^{*}p < 0.05$. Heteroskedasticity-robust standard errors.
The regressions are weighted based on matching on propensity scores from a logit model. Three types of matching strategies are used: either first ("KNN 1") or three first ("KNN 3") nearest neighbor(s), or using a rectangular kernel ("Kernel"). The regressions are run using the years 2018-2020.

Table SI.T17: Sample means balance before/after matching

ZDC attribution	Variable	Unweighted			Weighted		
		No ZDC	ZDC	Diff	No ZDC	ZDC	Diff
ZDC owner attribution	Average distance to first mill [km]	8.9	7.7	-1.2	8.0	7.6	-0.5
	Average concession area	91.8	115.8	24.0	105.7	106.5	0.7
	Average forest cover in 2000	32.2	32.2	-0.0	32.4	32.7	0.2
	Average deforestation 2001-12 (annualized)	7.1	7.1	-0.0	7.1	7.1	0.0
	Oil palm suitability index (%)	6359.9	6507.9	148.0	6591.2	6492.2	-99.0
ZDC buyer attribution	Average distance to first mill [km]	7.2	6.5	-0.7	6.8	6.2	-0.6
	Average concession area	75.7	104.7	29.0	90.4	89.6	-0.8
	Average forest cover in 2000	15.1	24.1	9.1	19.9	17.8	-2.1
	Average deforestation 2001-12 (annualized)	9.6	8.4	-1.1	7.7	8.1	0.4
	Oil palm suitability index (%)	7003.2	6666.0	-337.2	6758.3	6742.8	-15.4

Columns *unweighted* refer to the group means before matching, whereas columns *weighted* refer to the means after matching. Weighting was done by doing kernel matching on the propensity score estimated using a logit link.

Table SI.T18: Comparison of ZDC attribution based on owner/buyer score

Owner↓ / Buyer→	Linked to Undetermined	Linked to no ZDC	Linked to ZDC	Total Owner
Linked to Undetermined	270	9	66	345
Linked to no ZDC	159	14	61	234
Linked to ZDC	233	9	203	445
Total Buyer	662	32	330	1024

The numbers in the table indicate the number of concessions classified as *Linked to undetermined*, *No ZDC*, and *ZDC* based on both the owner-based and buyer-based attribution. The last row and last column indicate the total number of concessions based on each score, and therefore correspond to the numbers shown in Figure 2.

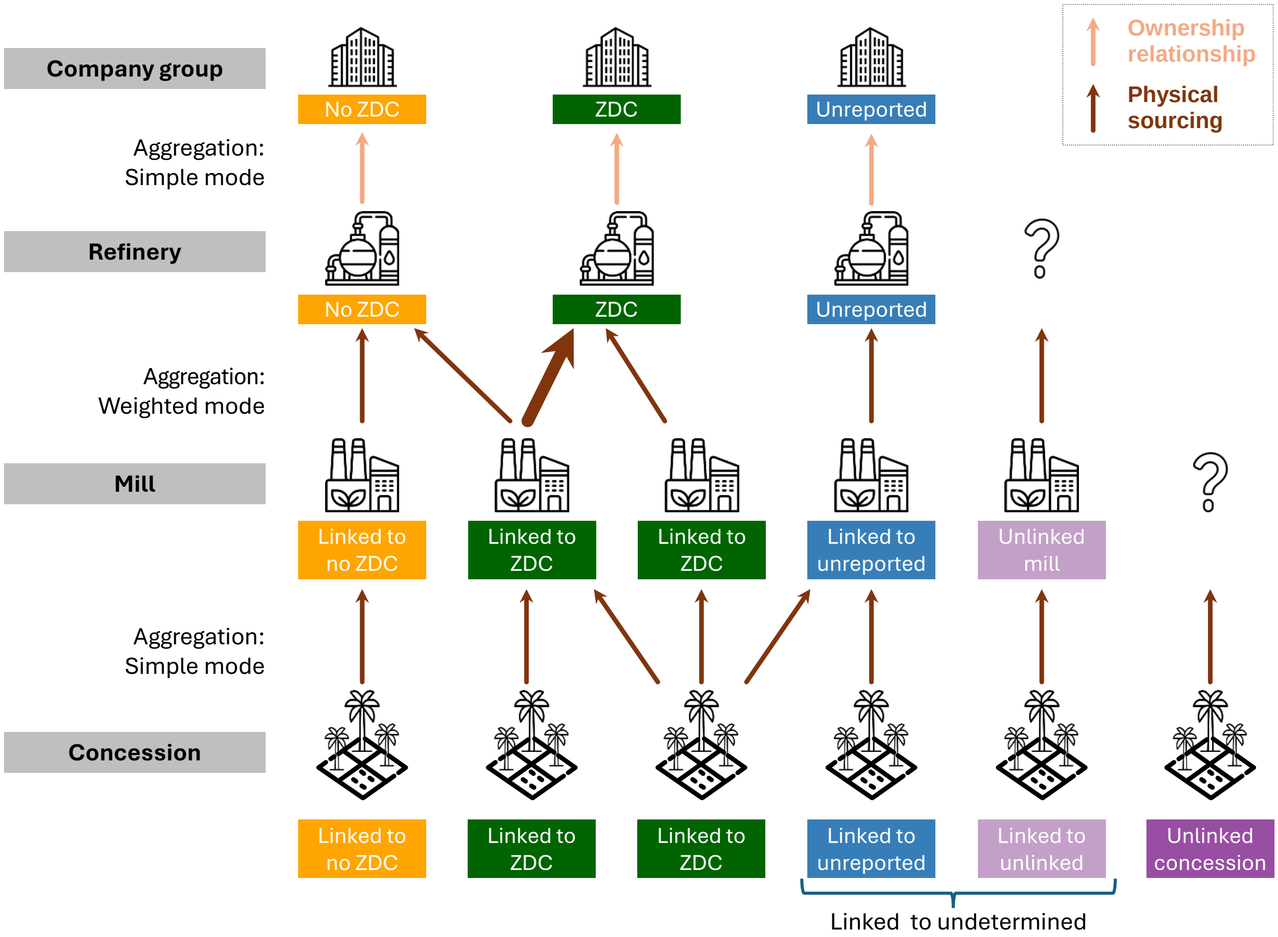

Figure SI.F9: Illustration of the sourcing-based attribution.

SI B Formalization of the DiD approach

In the main analysis, we use a difference-in-differences (DiD) approach comparing *post* and *pre*-intervention outcomes between concessions linked to ZDC companies (*treated*) against those not linked to ZDC companies (*control*). Unfortunately, we only observe the company-concession supply-chain linkages during the *post* period (2018-2020) and not during the *pre* period. Consequently, our DiD analysis defines the *treated* and *control* groups based on the *post* supply-chain linkages. However, some concessions that are part of ZDC in the *post* period might have been part of the non-ZDC supply chain in the *pre* period, or vice versa. Thus, we have four groups of concessions, the *persistently-linked-to-ZDC*, the *never-linked-to-ZDC*, the *newly-linked-to-ZDC* (linked to ZDC during the *post* but not *pre* period), and the *previously-linked-to-ZDC* (linked during the *pre* but not *post* period). Since we observe only the linkages to ZDC companies during the *post* treatment period, we cannot distinguish whether the treated units (concessions linked to ZDC in the *post* period) are *persistently-linked-to-ZDC* or *newly-linked-to-ZDC*, nor whether control units (concessions linked to no ZDC in the *post* period) are *never-linked-to-ZDC* or *previously-linked-to-ZDC*. In the following, we show that in this setting, the DiD estimator can be decomposed into three unobservable DiD estimators, corresponding to the DiD on *persistently-linked-to-ZDC*, on *newly-linked-to-ZDC*, and on *previously-linked-to-ZDC* concessions. We then show that under a "groupwise parallel trends" assumption, our DiD estimator identifies a weighted average of the causal effect on *persistently-linked-to-ZDC* and *newly-linked-to-ZDC* concessions. We also show how relaxing the groupwise parallel trends assumption towards an "adverse selection" assumption results in an upper bound on the causal effect on *persistently-linked-to-ZDC* and *newly-linked-to-ZDC* concessions.

To investigate this formally, we first introduce some notation to describe the four possible scenarios for a concession based on its linkage to the ZDC or non-ZDC supply chain during the *pre* and *post* periods. Concession groups are

denoted by the pairs YY, YN, NY, NN. The first letter (Y or N) indicates whether they were linked to a ZDC company during the *pre* period (Y for linked to ZDC, N for linked to non-ZDC), while the second letter represents their linkage to ZDC companies during the *post* period. The four groups of concessions are:

		Post period	
	ZDC:	Not linked to ZDC	Linked to ZDC
Pre period	Not linked to ZDC	**NN**: Never linked to ZDC	**NY**: Newly linked to ZDC
	Linked to ZDC	**YN**: Previously linked to ZDC	**YY**: Persistently linked to ZDC

- YY: **"Persistently linked to current ZDC companies"**: concessions that were linked both during the *pre* and *post* periods to companies that took ZDCs.
- NY: **"Newly linked to current ZDC companies"**: concessions that were not linked during the *pre* period, but were during the *post* period, to companies that took ZDCs.
- YN: **"Previously linked to current ZDC companies"**: concessions that were linked during the *pre* period, but not during the *post* period, to companies that took ZDCs.
- NN: **"Never linked to current ZDC companies"**: concessions that were never linked to companies that took ZDCs.

In our main analysis, we use what we call the **Post-supply-chain DiD (PSC-DiD)**, where we consider as treated/control the group of concessions that were linked/not-linked to ZDC companies during the *post* period. This means that we are considering as treated both *persistently-linked-to-ZDC* (YY) and *newly-linked-to-ZDC* (NY) concessions, and as control *previously-linked-to-ZDC* (YN) and *never-linked-to-ZDC* concessions (NN).

Definition 1 (Post-supply-chain DiD) *Let $DiD(\{A\};\{B\})$ denote the difference-in-differences estimator that uses units $\in \{A\}$ as treated, and units $\in \{B\}$ as control. We call **Post-supply-chain DiD**, and write $DiD^{PSC}(\{YY, NY\};\{NN, YN\})$, the DiD that uses post-treatment supply-chain status to define treated/control groups:*

$$DiD^{PSC}(\{YY, NY\};\{NN, YN\}) \equiv (\overline{Y}_{i\in\{YY,NY\},t\in post} - \overline{Y}_{i\in\{YY,NY\},t\in pre}) - (\overline{Y}_{i\in\{NN,YN\},t\in post} - \overline{Y}_{i\in\{NN,YN\},t\in pre})$$

Where Y is the outcome variable of interest (deforestation or forest cover) and $\overline{Y}$ denotes the sample average of Y.

We have the following decomposition result:

Theorem 1 (Decomposition of DiD^{PSC}) *The DiD^{PSC} estimates a combination of three unobservable DiDs:*

$$\begin{aligned} DiD^{PSC} \equiv& DiD(\{YY, NY\};\{NN, YN\}) \\ =& \pi_{YY} DiD(\{YY\};\{NN\}) + \pi_{NY} DiD(\{NY\};\{NN\}) - \pi_{YN} DiD(\{YN\};\{NN\}) \end{aligned} \tag{1}$$

Where $\pi_{YY} \equiv \frac{N_{YY}}{N_{YY}+N_{NY}}$ is the share of YY units among the units considered as treated ($\{YY, NY\}$). Likewise, $\pi_{NY} \equiv N_{NY}/(N_{YY} + N_{NY})$, and $\pi_{YN} \equiv N_{YN}/(N_{NN} + N_{YN})$.

Moreover, DiD^{PSC} can be decomposed into the following causal parameters:

$$\begin{aligned} DiD^{PSC} =& \pi_{YY} ATT(\{YY\}) + \pi_{NY} ATT(\{NY\}) \\ &+ \pi_{YY}\Delta_0(\{YY\};\{NN\}) + \pi_{NY}\Delta_0(\{NY\};\{NN\}) - \pi_{YN}\Delta_0(\{YN\};\{NN\}) \end{aligned} \tag{2}$$

Where:

- *$ATT(\{A\})$ represents the average treatment effect on the treated group $\{A\}$.*
- *$\Delta_0(\{A\};\{B\})$ denotes the "deviation from trends absent treatment", i.e. the difference in trends between group $\{A\}$ and group $\{B\}$ assuming that group $\{A\}$ is not treated during the* post *period, i.e. $\Delta_0(\{A\};\{B\}) \equiv \mathbb{E}_{i\in\{A\}}(Y(0)_{t\in post} - Y(0)_{t\in pre}) - \mathbb{E}_{i\in\{B\}}(Y(0)_{t\in post} - Y(0)_{t\in pre})$, where $Y(0)_{i,t}$ represents the potential outcome of unit i at time t if it is not treated.*

Equation [1] shows that the post-supply-chain DiD^{PSC} estimates a combination of three different unobservable DiDs:

- The DiD of **"persistently-linked-to-ZDC"** (YY) versus **"never-linked-to-ZDC"** (NN) concessions, i.e. $DiD(\{YY\};\{NN\})$
- The DiD of **"newly-linked-to-ZDC"** (NY) versus **"never-linked-to-ZDC"** (NN) concessions, i.e. $DiD(\{NY\};\{NN\})$
- The DiD of **"previously-linked-to-ZDC"** (YN) versus **"never-linked-to-ZDC"** (NN) concessions, i.e. $DiD(\{YN\};\{NN\})$.

Equation [2] shows how the three underlying DiD can be written in terms of causal effects ATT and deviations from trends Δ_0. Importantly, there is no causal effect attached to the last DiD on *previously-linked-to-ZDC* ($DiD(\{YN\};\{NN\})$) since there is no treated unit among *previously-linked-to-ZDC* concessions (units were either linked to ZDC but before treatment, or not linked to ZDC after treatment). We show here two different sets of assumptions under which the PSC-DiD has a clear causal interpretation.

Assumption 1 (Groupwise parallel trends) *Assume that every subgroup* $\{YY, NY, YN\}$ *is parallel with respect to the* never-linked-to-ZDC *(*$\{NN\}$*) subgroup absent treatment. That is:* $\Delta_0(\{YY\};\{NN\}) = \Delta_0(\{NY\};\{NN\}) = \Delta_0(\{YN\};\{NN\}) = 0$*. Then:*

$$DiD^{PSC} = \pi_{YY} ATT(\{YY\}) + \pi_{NY} ATT(\{NY\})$$

Assumption 2 (Selection on trends) *Assume that the ZDC companies selectively kept or included those concessions that were anyway doing better than the reference group* $\{NN\}$*, and selectively discarded those concessions that were doing worse than the reference group* $\{NN\}$*. That is:* $\Delta_0(\{YY\};\{NN\}) \geq 0$*,* $\Delta_0(\{NY\};\{NN\}) \geq 0$ *and* $\Delta_0(\{YN\};\{NN\}) \leq 0$*. Then:*

$$DiD^{PSC} \geq \pi_{YY} ATT(\{YY\}) + \pi_{NY} ATT(\{NY\})$$

Under the "Groupwise parallel trends" assumption 1, every subgroup is assumed to be parallel, absent treatment, to the "never-linked-to-ZDC" subgroup (NN), i.e. $\Delta_0(A, \{NN\}) = 0 \; \forall A \in \{YY, NY, YN\}$. Under this condition, the post-supply-chain DiD^{PSC} identifies a composite effect $\pi_{YY} ATT(\{YY\}) + \pi_{NY} ATT(\{NY\})$. This is a well-defined parameter that is a weighted mean of the average treatment effect on the treated (ATT) of the "persistently-linked-to-ZDC" (YY) and "newly-linked-to-ZDC" (NY) groups, where the weights π_{YY} and π_{NY} represent the share of each subgroup, with $\pi_{YY} + \pi_{NY} = 1$. Under the ownership-based ZDC linkages, where ownership changes slowly over time and therefore π_{NY} is likely small, DiD^{PSC} likely identifies a parameter close to the ATT for the "persistently-linked-to-ZDC" (YY). On the other hand, for the sourcing-based ZDC linkages, where changes happen more often, DiD^{PSC} likely identifies an effect representative of the ATT on both the "persistently-linked-to-ZDC" (YY) and the "newly-linked-to-ZDC" (NY).

The "Groupwise parallel trends" assumption 1, is stronger than the usual parallel trends assumption as it requires not only that the whole *treated* group be parallel to the *control* group, absent treatment, but that this holds for (unobserved) subgroups. It also implies that "persistently-linked-to-ZDC", "newly-linked-to-ZDC" and "previously-linked-to-ZDC" would also be parallel to each other, absent treatment. This basically assumes that ZDC companies do not selectively choose concessions based on differential trends.

Assumption 2 shows that the "Groupwise parallel trends" can be relaxed by allowing selection into the treatment. We assume here an *adverse selection* scenario under which companies select the concessions they own/source from in order to clean their supply chain, that is : 1) concessions that would be doing better than the *never-linked-to-ZDC* are either included (NY) or kept (YY) in the ZDC supply-chain, 2) ZDC concessions that would be doing worse than the *never-linked-to-ZDC* are excluded from ZDC (YN). Under this scenario, assumption 2 shows that the DiD^{PSC} provides an upper bound on the composite effect. To illustrate it, assume that the outcome variable is forest cover. Adverse selection implies that concessions that were conserving more forest become ZDC, $\Delta_0(\{NY\};\{NN\}) > 0$, and that those that were conserving less become non-ZDC, $\Delta_0(\{YN\};\{NN\}) < 0$. Then we have $DiD^{PSC} \geq \pi_{YY} ATT(\{YY\}) + \pi_{NY} ATT(\{NY\})$. Conversely, when the outcome variable is deforestation, we have $\Delta_0(\{NY\};\{NN\}) < 0$ and $\Delta_0(\{YN\};\{NN\}) > 0$. This implies that $DiD^{PSC} \leq \pi_{YY} ATT(\{YY\}) + \pi_{NY} ATT(\{NY\})$, and thus that the DiD is overstating deforestation reductions.

A further complication arises from the fact that the treatment status is not observed during the "partial ZDC implementation" period. The same reasoning as above can be applied, using now three letters to represent the pre-, partial-, and full-implementation statuses. For simplicity, suppose there are only four groups: YYY, YYN, NNN, and NNY. The notation follows the same logic as before: NNY, for instance, refers to a concession that was not linked during the *pre* and *partial* periods but became linked to ZDCs in the *full* period. In that case, Equation (1) can be rewritten as follows under the groupwise parallel trend assumption: $DiD(\{YYY, NNY\}; \{NNN, YYN\}) = \pi_{YYY}ATT(\{YYY\}) + \pi_{NNY}ATT(\{NNY\}) - \pi_{YYN}ATT(\{YYN\})$. Unlike the previous case, where $DiD(\{YN\}, \{NN\})$ was zero due to the absence of any treatment for YN units, the term $DiD(\{YYN\}, \{NNN\})$ now remains in the equation due to the presence of partially-treated units YYN. As a consequence, $DiD(\{YYY, NNY\}; \{NNN, YYN\})$ will be larger than the parameters of interest, the effect of ZDC treatment during the *full* implementation period, $\pi_{YYY}ATT(\{YYY\}) + \pi_{NNY}ATT(\{NNY\})$.

Proof of Theorem (1):

Let us denote by $\Delta(\{A\})$ the time difference of average post-pre outcomes Y for group $\{A\}$:

$$\Delta(\{A\}) \equiv (\overline{Y}_{i\in\{A\},t\in post} - \overline{Y}_{i\in\{A\},t\in pre})$$

Note that, if group A consists of two subgroups, A' and A'', then:

$$\Delta(\{A', A''\}) = \pi_{A'}\Delta(\{A'\}) + \pi_{A''}\Delta(\{A''\})$$

where $\pi_{A'}$ is the share of A' among A' and A'', i.e. $\pi_{A'} = N_{A'}/(N_{A'} + N_{A''})$. This comes from the simple fact that the mean of a group is equal to the weighted mean of the subgroups, i.e. $\overline{Y}_{i\in\{A',A''\}} = \pi_{A'}\overline{Y}_{i\in\{A'\}} + \pi_{A''}\overline{Y}_{i\in\{A''\}}$.

$$\begin{aligned}
DiD^{PSC}(\{YY, NY\}; \{NN, YN\}) \equiv& \Delta(\{YY, NY\}) - \Delta(\{NN, YN\}) \\
=& \pi_{YY}\Delta(YY) + \pi_{NY}\Delta(NY) - \pi_{YN}\Delta(YN) - \pi_{NN}\Delta(NN) \\
=& \pi_{YY}\left(\Delta(YY) - \Delta(NN)\right) + \pi_{NY}\left(\Delta(NY) - \Delta(NN)\right) - \pi_{YN}\left(\Delta(YN) - \Delta(NN)\right) \\
&+ (\pi_{YY} + \pi_{NY} - \pi_{NN} - \pi_{YN})\Delta(NN) \\
=& \pi_{YY}DiD(\{YY\}; \{NN\}) + \pi_{NY}DiD(\{NY\}; \{NN\}) - \pi_{YN}DiD(\{YN\}; \{NN\})
\end{aligned}$$

SI C Supplementary details on data assembly

SI C.1 Data selection rules

Concessions were selected based on the following sequential criteria for the econometric analysis:

1. We only kept concessions that were in the (groups of) islands of Kalimantan, Papua, Sulawesi and Sumatra, excluding therefore concessions in Java (126) and Maluku (20). For Java, this was motivated by the fact that concessions in Java are likely to be relatively old and hold little remaining forest. For Maluku, this was motivated by the fact that there are very few concessions and furthermore only a single mill on that group of islands, so that there would not be any variation in the ZDC status of the concessions.

2. We then only kept concessions that had some initial forest cover in 2000, given that no further deforestation is possible on these concessions and that annualized deforestation rates are not well defined in this case (division by zero).

3. We then only kept concessions that had experienced some oil-palm driven deforestation according to Gaveau et al. (4). This was motivated by the fact that concessions where no deforestation occurred from 2001 to 2020 might not represent active concessions. We test the sensitivity of our results to this decision in Table SI.T12.

   We then only kept concessions that were linked to a mill[1] and received a status of *linked to no ZDC* or *linked to ZDC*, using the *unlinked* and *Linked to undetermined* as control groups in robustness tests (see Table SI.T4).

[1] Note that unlinked concessions are identical under the owner- and buyer-based attributions, since the underlying set of mills is the same. In contrast, the set of unlinked mills differs across the two attributions, as mills are compared against different sets of company groups.

The main reasons to focus on the *no ZDC* as control group in the main analysis were due to the increased confidence in the ZDC status of this group (given that *unlinked* and *Linked to undetermined* cannot be given a ZDC score due to absent linkage information) as well as the closer behavior of this group compared to the treatment group (see Table SI.T1 and Figure SI.F4).

Table SI.T19 indicates the number of units removed (*N remove*) at each step. Note that this number is highly dependent on the ordering of the sequential rules, which is mainly arbitrary. For the final econometric analysis, we kept the concessions that were given an ownership/sourcing-based ZDC status of *ZDC* or *no-ZDC*, discarding those that had either a status of *Unlinked* or *Linked to undetermined.* In a robustness analysis, we use instead the *unlinked* or *linked to undetermined* groups as control, see SI.T4.

Table SI.T19: Selection rules for concessions.

Rule type	Selection Rule	N Initial	N Remove	N Final
Sequential rule	Is concession in a main island?	2644	146	2498
	Does concession have initial forest > 0?	2498	178	2320
	Does concession contain industrial palm oil?	2320	992	1328
	Is concession linked to a mill?	1328	584	744
Alternative rule	Is concession's linked mill itself linked:			
	- to a known mill-owning company (owner attribution)?	744	252	492
	- to a known refining company (buyer attribution)?	744	513	231

The table indicates the number of concessions before the selection rule is applied (*N initial*), the number of units affected by the selection rule (*N Remove*), and the remaining number of units once the rule has been applied (*N Final*).

SI C.2 Clustering procedure

Our goal here is to categorize concessions according to their stage of frontier development, defined by the temporal evolution in terms of initial forest cover and subsequent deforestation during the pre-ZDC period. To cluster the concessions according to their frontier stage, we applied a k-means algorithm using forest cover in 2000 and 2010 as clustering variables. Both variables were scaled to have unit variance. After careful graphical examination, we opted to keep three clusters. This decision was made to facilitate interpretation and to ensure that each cluster contained a sufficient number of observations. Table SI.T20 shows the means for each cluster. We interpreted the three clusters as follows:

1. "Old frontier, inactive by 2010", noting that the forest cover is low both in 2000 and 2010.
2. "New frontier, active in 2010", noting that there is a relatively high forest cover in 2000 and a large decline in forest cover by 2010.
3. "New frontier, inactive in 2010", noting that there are high levels of initial forest cover in 2000 and a small decline by 2010.

Table SI.T21: Details on the components retained for assessing corporate ZDC quality and criteria adapted from SPOTT indicators

Principle categories	Included SPOTT criteria
**Conservation commitment**	
ZDCs should be stringent with clear targets, geographical scope, and commitment on conserving remaining forest cover, preventing forest conversion, and restoration commitments.	1. Conservation commitment includes a pledge on deforestation- and forest conversion-free supply chain<br>2. Commitment to not planting on peatland.
**Traceability commitment**	
ZDCs should have a time-bound commitment to achieve traceability to both mill and plantation levels for their own and supplier's plantations to enhance transparency and prevent spillover.	1. Timebound commitment to achieve full traceability to mill<br>2. Timebound commitment to achieve full traceability to plantation<br>3. *Traceability implementation to mills*<br>4. *Traceability implementation, from own mills to plantation.*
**Smallholder inclusion**	
ZDC should provide program aimed to support smallholders, for example via capacity building, that are differentiated for different types of smallholders with a clear informed target.	1. Design program to support scheme/plasma smallholders<br>2. Design program to support independent smallholders<br>3. *Participation of independent or plasma smallholder involvement in the programme*
**Compliance commitment**	
ZDCs should provide clear and functional assessment and engagement mechanisms.	1. Program to support high-risk mills (at high risk of contributing to deforestation) to become compliant.<br>2. *Regularly engages with high-risk mills*
**Monitoring commitment**	
ZDCs should provide reliable and frequent monitoring systems.	1. Available and open grievance or complaint system<br>2. *Self-reported evidence of monitoring deforestation*
**Transparency**	
ZDCs should allow for transparency in their enforcement approach.	1. Disclosing the details of complaints and grievances, which includes the action and enforcement status.

Criteria in *italics* pertain to the progress of commitment implementation by companies, which will be included in analyzing the quality of companies' ZDC.

Table SI.T20: Cluster means.

ZDC aggregation method	Cluster number	ZDC score	N units	Forest cover 2000	Forest cover 2010	2000-2010 forest cover change
ZDC owner attribution	1	Linked to no ZDC	91	11.9	4.7	7.2
	1	Linked to ZDC	201	9.8	4.1	5.7
	2	Linked to no ZDC	53	48.3	17.8	30.4
	2	Linked to ZDC	97	47.7	18.8	29.0
	3	Linked to no ZDC	20	69.0	59.3	9.7
	3	Linked to ZDC	30	75.9	69.1	6.9
ZDC buyer attribution	1	Linked to no ZDC	18	9.0	2.9	6.1
	1	Linked to ZDC	126	7.9	2.8	5.0
	2	Linked to no ZDC	7	32.4	15.0	17.4
	2	Linked to ZDC	65	43.3	13.9	29.3
	3	Linked to no ZDC	2	56.0	49.6	6.4
	3	Linked to ZDC	13	69.9	53.1	16.8

The table indicates various statistics for each cluster as defined above. *N units* indicates the number of concessions in each cluster, *Forest cover 2000* and *2010* indicate the mean forest cover (in %, with respect to the concession total area) in each cluster, whereas *2000-2010 forest cover change* indicates the difference in forest cover between 2000 and 2010.

References

[1] Yiqing Xu. Generalized synthetic control method: Causal inference with interactive fixed effects models. *Political Analysis*, 25(1):57–76, January 2017. ISSN 1476-4989. doi: 10.1017/pan.2016.2.

[2] Susan Athey, Mohsen Bayati, Nikolay Doudchenko, Guido Imbens, and Khashayar Khosravi. Matrix completion methods for causal panel data models. *Journal of the American Statistical Association*, 116(536):1716–1730, May 2021. ISSN 1537-274X. doi: 10.1080/01621459.2021.1891924.

[3] Thomas J. Leeper. *margins: Marginal Effects for Model Objects*, 2024. URL https://cran.r-project.org/package=margins. R package version 0.3.28.

[4] David L. A. Gaveau, Bruno Locatelli, Mohammad A. Salim, Husnayaen, Timer Manurung, Adrià Descals, Arild Angelsen, Erik Meijaard, and Douglas Sheil. Slowing deforestation in Indonesia follows declining oil palm expansion and lower oil prices. *PLOS ONE*, 17(3):e0266178, March 2022. ISSN 1932-6203. doi: 10.1371/journal.pone.

0266178. URL https://journals.plos.org/plosone/article?id=10.1371/journal.pone.0266178. Publisher: Public Library of Science.
